# Crystal Structures and Phase Evolutions of Potassium Decahydro-*closo*-Decaborate and Decahydro-1-Carba-*closo*-Decaborate: K_2_B_10_H_10_ and K-1-CB_9_H_10_

**DOI:** 10.1021/acs.inorgchem.6c01850

**Published:** 2026-07-14

**Authors:** Hui Wu, Wei Zhou, Benjamin A. Trump, Craig M. Brown, Terrence J. Udovic

**Affiliations:** † NIST Center for Neutron Research, 10833 National Institute of Standards and Technology, Gaithersburg, Maryland 20899-6102, United States; ‡ Department of Materials Science and Engineering, University of Maryland, College Park, Maryland 20742, United States; § Intel Gordon Moore Park at Ronler Acres, Hillsboro, Oregon 97124, United States

## Abstract

The ordered crystal structures and phase evolutions of potassium decahydro-*closo*-decaborate (K_2_B_10_H_10_) and monocarba-*closo*-decaborate (K-1-CB_9_H_10_) were investigated by using synchrotron X-ray diffraction in combination with first-principles calculations and neutron vibrational spectroscopy. Upon cooling from room temperature to low temperature (100 K), both compounds exhibit order-to-order structure transitions with two ordered K_2_B_10_H_10_ and three ordered K-1-CB_9_H_10_ structures being identified and solved. For K_2_B_10_H_10_, a gradual monoclinic to monoclinic phase transition is observed. In contrast, KCB_9_H_10_ undergoes a more abrupt first-order tetragonal-to-orthorhombic phase transition. Our detailed structural analyses indicate that these transitions are driven by the reorientation of the quasi-ellipsoidal anions, optimizing the anion packing efficiency and cation–anion interactions. Notably, the reduced cation stoichiometry and weaker cation–anion interactions in KCB_9_H_10_ lead to significantly lower anion reorientational barriers compared with K_2_B_10_H_10_. Uncovering the orientational flexibility of these quasi-ellipsoidal anions at low temperatures is essential for understanding their dramatic transformation into superionic conductors at elevated temperatures. This work provides the structural foundation necessary for designing and developing next-generation polyborate-based solid-state electrolytes.

## Graphical abstract

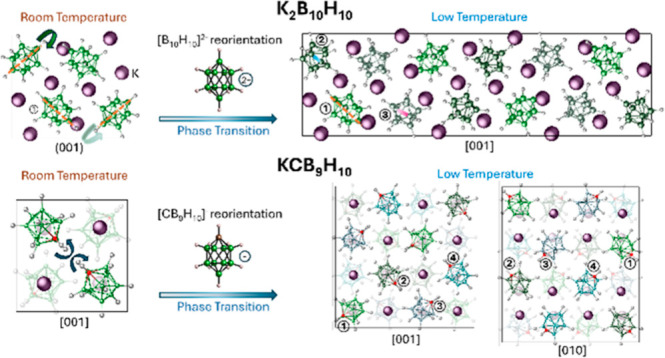

## Introduction

Alkali-metal polyborate compounds containing the relatively stable dodecahydro-*closo*-dodecaborate ([B_12_H_12_]^2–^),^1,2^ decahydro-*closo*-decaborate ([B_10_H_10_]^2–^) anions,^3^ and their related *closo*-monocarbaborate ([CB_11_H_12_]^−^ and [1-CB_9_H_10_]^−^)^4,5^ and *nido*-poly­(carba)­borate ([B_11_H_14_]^−^, [7-CB_10_H_13_]^−^, [7,8-C_2_B_9_H_12_]^−^, [7,9-C_2_B_9_H_12_]^−^)^6,7^ anions have attracted increasing attention for their great promise as superionic electrolytes for next-generation solid-state rechargeable batteries. Some of these compounds such as ACB_11_H_12_ and A-1-CB_9_H_10_ (A = Li and Na) in their disordered-state polymorphs can exhibit liquid-like superionic conductivities that match or surpass the benchmark polycrystalline Li^+^ and Na^+^ electrolytes near or at room temperature.^4,5,8–11^ Many structural and dynamic studies of these materials have revealed a sudden jump in ionic conductivity accompanying a phase transition from an ordered room-temperature (RT) or low-temperature (LT) (i.e., below RT) phase to an entropically driven, disordered high-temperature (HT) phase (i.e., above RT).^1,3–7^ In the crystal structures of the HT disordered phases of these compounds, the large polyhedral anions are orientationally disordered and can be viewed as quasi-spheres. They often arrange themselves in a typical close-packing scheme of equal spheres such as face-centered-cubic (*fcc*) A_2_B_12_H_12_ (A = Li, Na, K, Rb, Cs),^12–15^ Na_2_B_10_H_10_,^3^ ACB_11_H_12_ (A = Li, Na, K, Rb, and Cs),^4,16,17^ AB_11_H_14_(A = Li and K),^7,14^ A-7-CB_10_H_13_(A = Na, K),^6,7^ A-7,9-C_2_B_9_H_12_ (A = Na, K),^6,7^ and K-7,8-C_2_B_9_H_12_;^6,7^ hexagonal-close-packed (*hcp*) A-1-CB_9_H_10_ (A = Li and Na),^5^ Na-7,8-C_2_B_9_H_12_,^6^ K-7,9-C_2_B_9_H_12_,^7^ and ACB_11_H_12_ (A = Na and Cs);^4,16^ and body-centered-cubic (*bcc*) NaB_11_H_14_ ^6^ and Na_2_B_12_H_12_.^1,12^ A disordered alkali-metal polyborate sometimes can also exhibit several polymorphs with different anion close-packing schemes such as Na_2_B_12_H_12_ (*fcc* and *bcc*),^18^ NaCB_11_H_12_ (*fcc*, *hcp* and *bcc*),^4^ K-7,9-C_2_B_9_H_12_ (*fcc* and *hcp*),^7^ and CsCB_11_H_12_ (*fcc* and *hcp*)^16^ at different high temperatures. These anion arrangements would create spacious cation diffusion corridors within the sublattice of the large disordered quasi-spherical anions.

Compared to the handful of crystal structural types known for the disordered HT phases of these polyborates, the crystal structures of their ordered RT and LT phases, where cations and anions have well-defined positions and orientations, are diverse. The RT- and LT-ordered crystal structures of almost all stable crystalline phases of alkali-metal dodecahydro-*closo*-dodecaborates (A_2_B_12_H_12_, A = Li, Na, K, Rb, and Cs)^14,15,18–20^ and their closely related *closo*-carbaborate counterparts (ACB_11_H_12_, A = Li, Na, K, and Cs),^4,16,17^ as well as some *nido*-(carba)­borates (AB_11_H_14_, A-7-CB_10_H_13_, A-7,8-C_2_B_9_H_12_, A-7,9-C_2_B_9_H_12_, A = Na, K)^6,7^ with potentially interesting ionic conductivity properties in their HT phases, have been well investigated. In contrast, thorough studies on the ordered structures of alkali-metal decahydro-*closo*-decaborates and their related *closo*-monocarbaborates (A_2_B_10_H_10_ and A-1-CB_9_H_10_) are limited and still awaiting completion. Hofmann et al. first reported that alkali-metal decahydro-*closo*-decaborates (A_2_B_10_H_10_ A = Na, K, and Rb)^21^ possess an ordered monoclinic structure at room temperature using powder X-ray diffraction (XRD). However, they found that only Na_2_B_10_H_10_ can maintain the same structure after cooling to low temperatures, while a phase transition in K_2_B_10_H_10_ and loss of crystallinity in Rb_2_B_10_H_10_ below 273 K prevented the determination of their LT structures. Later, we successfully determined the RT and LT structures of Li_2_B_10_H_10_ ^22^ and further revised the previously reported crystal structure of Na_2_B_10_H_10_ using neutron powder diffraction (NPD).^23^ More recently, we also studied the structural variations of the ordered Rb_2_B_10_H_10_ in the temperature range of 100 K–600 K and found it exhibits a monoclinic-to-triclinic phase transition.^24^ This transition should be responsible for the origin of the crystallinity loss of Rb_2_B_10_H_10_ at low temperatures mentioned in the literature.

As for the crystal structures of the alkali metal *closo*-monocarbaborate analogues (A-1-CB_9_H_10_), when one of the apical B–H vertices of the [B_10_H_10_]^2–^ anion is replaced by the C–H group, the bivalent B_10_H_10_^2–^ anion is reduced to the monovalent [CB_9_H_10_]^−^ anion. This substitution would directly result in significant structural consequences: (i) only half as many A^+^ cations as in the A_2_B_10_H_10_ structure; (ii) weaker Coulombic interactions between anions and their surrounding cations, and (iii) charge polarization of the anions.^5,25^ Therefore, although the [1-CB_9_H_10_]^−^ anion still maintains the bicapped-square-antiprismatic shape of the [B_10_H_10_]^2–^ anion, one cannot assume that the same ordered structures of A_2_B_10_H_10_ would apply to A-1-CB_9_H_10_. We have conducted detailed temperature-dependent X-ray structural studies of LiCB_9_H_10_ and NaCB_9_H_10_ and have shown that these monocarbaborates indeed possess different ordered structures compared with their decaborate counterparts.^5,26^ These structural consequences induced by C–H replacement have also been shown to promote a reduced order–disorder phase-transition temperature (*T*_trans_) and faster anion reorientations in the disordered ACB_9_H_10_ (A = Li, Na) compared to their A_2_B_10_H_10_ counterparts.^5,25^

As we have demonstrated in our previous works on lithium (Li), sodium (Na), and rubidium (Rb) decahydro-*closo*-decaborates and their *closo*-monocarbaborate cousins (i.e., Li-1-CB_9_H_10_ and Na-1-CB_9_H_10_),^4,22–24,26^ the structural determination of alkali-metal polyborates is nontrivial. This often requires first-principles calculations to confirm the stability of any proposed structural model and additional vibrational spectroscopy to corroborate powder X-ray diffraction in the absence of complementary neutron diffraction data. These techniques are necessary to validate the proposed structural model, especially when multiple energy minima are possible. Therefore, it would be worthwhile to undertake the endeavor to solve the puzzle of K_2_B_10_H_10_ and KCB_9_H_10_ structures so as to further our goal to complete the whole set of crystal structures for alkali-metal decahydro-*closo*-decaborates and their related *closo*-monocarbaborates. Moreover, the determined crystal structures of these compounds will help researchers to better understand their temperature-dependent structural behaviors, phase transitions, and thermodynamic properties, as well as to perform meaningful computational studies to correlate with the anion dynamics and possible superionic conduction in the structurally disordered HT phases.

In the present study, we focus on the ordered structures of K_2_B_10_H_10_ and KCB_9_H_10_. We identified and determined two ordered structures of K_2_B_10_H_10_ and three ordered structures of KCB_9_H_10_ using synchrotron X-ray powder diffraction (XRPD) combined with first-principles calculations and corroborated them by neutron vibrational spectroscopy (NVS). Phase transitions and temperature-dependent structural behaviors of these ordered structures were also investigated and discussed based on the results of in situ synchrotron XRPD and differential scanning calorimetry (DSC).

## Experimental Section

Potassium decahydro-*closo*-decaborate (K_2_B_10_H_10_) and potassium decahydro-1-carba-*closo*-decaborate (K-1-CB_9_H_10_) were obtained from Katchem. (N.B., as there are two possible monocarba-isomers, 1-carba- refers to carbon occupying an apical position; 2-carba- to carbon occupying one of the eight equatorial positions of the bicapped-square-antiprismatic CB_9_H_10_^–^ anion. Throughout this paper, we only investigated the salt of the 1-carba-isomer form, henceforth referred to simply as KCB_9_H_10_.) Both K_2_B_10_H_10_ and KCB_9_H_10_ hygroscopic salts were vacuum-annealed at 453 K for about 16 h to ensure full dehydration prior to investigations.

Synchrotron XRPD patterns of K_2_B_10_H_10_ and KCB_9_H_10_ in sealed quartz capillaries were, respectively, measured between 138 and 480 K and between 100 and 298 K at the Advanced Photon Source on Beamline 17-BM-B at the Argonne National Laboratory using a Si(111) monochromator (λ = 0.45236(1) Å and 0.45415(1) Å, respectively). Neutron vibrational spectroscopy (NVS) measurements were performed at the National Institute of Standards and Technology Center for Neutron Research on the Filter-Analyzer Neutron Spectrometer (FANS)^27^ using the Cu(220) monochromator with pre- and post-collimations of 20′ of arc, yielding a full-width-at-half-maximum (fwhm) energy resolution of about 3% of the neutron energy transfer. Differential scanning calorimetry (DSC) measurements were conducted with a Netzsch (STA 449 *F*1 Jupiter) TGA-DSC under He flow using finely ground samples in cold-weld-sealed Al sample pans with a heating/cooling rate of ±20 K·min^–1^. All sample handling, storage, and sealing procedures were performed under a helium protective atmosphere or within a helium-filled glovebox.

The crystal structures of K_2_B_10_H_10_ and KCB_9_H_10_ were first solved using direct space methods with Fox program.^28^ Density functional theory (DFT) calculations were subsequently performed to optimize the B_10_H_10_^2–^ and CB_9_H_10_^–^ configurations and K positions in the initially solved structural models, followed by Rietveld refinement of the DFT-relaxed structural models using the GSAS package.^29,30^ The DFT-optimized B_10_H_10_^2–^ and CB_9_H_10_^–^ anions were kept as rigid bodies during refinement in order to maintain the integrity of anion geometry and a stable refinement due to the relatively poor X-ray sensitivity for H and the significantly large number of refinable variables (e.g., three crystallographically distinct B_10_H_10_^–^ anions and six K^+^ cations in the low-temperature monoclinic structure of K_2_B_10_H_10_ will generate [(3 × 20) + 6] × 3 atomic coordinate parameters). The coordinates of the anion rigid bodies and K^+^ cations together with the lattice parameters were refined. Thermal factors of like atoms were constrained to be the same. The magnitudes of thermal parameters from Rietveld refinement on XRD powder data are notoriously inaccurate. They are listed in the CIFs but we do not ascribe any significance to their values.

To assist and complement the structural refinements and NVS measurements, first-principles calculations were performed within the plane-wave implementation of the generalized gradient approximation to DFT using a Vanderbilt-type ultrasoft potential with Perdew–Burke–Ernzerhof exchange correlation.^31^ A cutoff energy of 544 eV and a 2 × 2 × 2 *k*-point mesh (generated using the Monkhorst–Pack scheme) were used and found to be enough for the total energy to converge within 0.01 meV/atom. For comparison with the NVS measurements, the phonon density of states (PDOS) was calculated for the DFT-optimized 0 K K_2_B_10_H_10_ and KCB_9_H_10_ structures using the supercell method (1 × 2 × 2 and 1 × 1 × 1 cell size, respectively) with finite displacements^32,33^ and were appropriately weighted to take into account the H, K, B, and C total neutron scattering cross sections. In addition, the PDOSs of the isolated B_10_H_10_^2–^ and 1-CB_9_H_10_^–^ anions were calculated for comparison using the 20 × 20 × 20 and 30 × 30 × 30 supercells and considering their D_4d_ and C_4v_ molecular symmetries in the phonon calculations, respectively. Both one-phonon and two-phonon contributions were calculated. Note that under the harmonic approximation, the dynamic structure factor can be expanded as a power series, yielding distinct terms for Bragg, one-phonon, and multiphonon scattering. Within the incoherent approximation, the two-phonon cross section simplifies to a self-convolution of the generalized phonon density of states.^34^

For all figures, standard uncertainties are commensurate with the observed scatter in the data if not explicitly designated by vertical error bars.

## Results

### Crystal Structures of K_2_B_10_H_10_

The room-temperature XRD pattern of K_2_B_10_H_10_ can be satisfactorily fit using a monoclinic structural model in space group *P2*_1_ */n* (Figure 1a) with refined lattice parameters of *a* = 12.857(1) Å, *b* = 11.1874(9) Å, *c* = 6.8279(5) Å, β = 93.302(4)°, and V = 980.5(2) Å^3^. This refined structure is consistent with the reported K_2_B_10_H_10_ structure^21^ at room temperature (RT phase or RT structure hereafter), where the decahydro-*closo*-decaborate cages have a well-defined orientation and arrange themselves in a slightly distorted hexagonal-close-packed (*hcp*) fashion characterized by an···ABABAB··· stacking sequence where alternate layers align identically (Figure S1). The K^+^ cations occupy the tetrahedral interstitial positions defined by four B_10_H_10_^–^ anions from two neighboring *ac* planes (Figure S2). Each anion is surrounded by 12 closest neighboring anions, and the distances between the mass centers of the anions range from 6.64 Å to 7.72 Å (mean 7.08 Å). This monoclinic RT structure remains stable up to 480 K (Figure S3).

**Figure 1:**
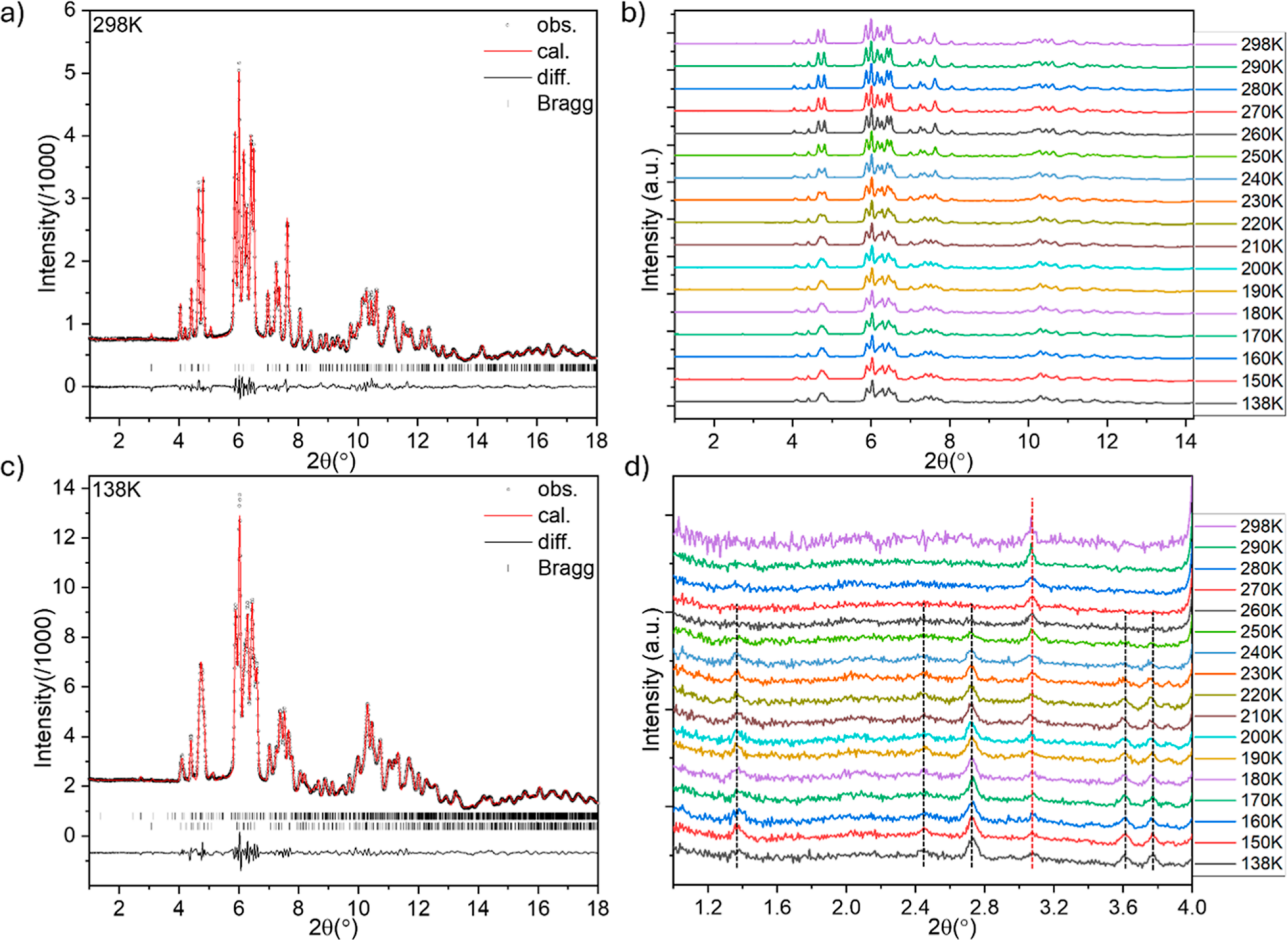
(a) Experimental (circles), fitted (line), and difference (line below observed and calculated patterns) synchrotron XRD profiles for K_2_B_10_H_10_ at 298 K. Vertical bars indicate the calculated positions of Bragg peaks. *R*_wp_ = 0.0401, *R*_p_ = 0.0286, χ^2^ = 1.17; (b) temperature-dependent XRD patterns indicating the phase evolution from 298 to 138 K (an enlarged view is shown in Figure S5); and (c) experimental (circles), fitted (line), and difference (line below observed and calculated patterns) synchrotron XRD profiles for K_2_B_10_H_10_ at 138 K. Vertical bars indicate the calculated positions of Bragg peaks of the LT phase (60.0(1) wt %, top) and *RT* phase structures (40.0(1) wt %, bottom). *R*_wp_ = 0.0325, *R*_p_ = 0.0238, χ^2^ = 1.626; (d) evolution of the diffraction peaks in the low-angle 2θ range of the temperature-dependent XRD patterns with a phase transition starting at about 260 K. Black dashed lines indicate the emerging diffraction peaks from the LT phase, and the red dashed lines show the dwindling diffraction peaks from the *RT* phase with decreasing temperature. XRD measurement wavelength λ = 0.45236 Å.

Upon cooling from room temperature to 138 K, temperature-dependent (T-dependent) synchrotron powder XRD patterns clearly indicated a gradual phase transition beginning at approximately 260 K. Figures 1b,d, S4a and S5 illustrate the variation of XRD patterns with temperature and the evolution of the new low-temperature phase (LT phase or LT structure hereafter). Careful inspection revealed that the new peaks from the LT phase seemed to cease evolving around 200 K, and further decreasing the temperature only resulted in the peak shifting caused by thermal contraction. Meanwhile, most diffraction peaks from the RT phase persisted at all studied temperatures, and their intensities also stopped changing below approximately 200 K. To complicate matters, except for only several satellite superlattice peaks distinctly present in the low-angle range, most of the peaks from the newly emerged LT phase largely overlap those of the RT phase (Figure S4). The positions of these overlapped peaks from the LT and RT phases move together with a decrease in temperature due to thermal contraction.

The overlapped main peaks of the LT phase with those of the RT phase suggest that this unknown LT phase might possess a similar monoclinic unit cell as the RT structure. When considering that the *d*-spacings of the distinct superlattice peaks extended to the low angle range of the LT phase XRD patterns (Figure 1d), the diffraction peaks of the LT phase can be best indexed into a much larger monoclinic unit cell with a tripled dimension 3*a*_RT_ × *b*_RT_ × *c*_RT_ and a similar β angle. The LT crystal structure (coexisting with the RT structure) was then solved based on this unit cell with space group *P*2_1_/*n* using the 138 K XRD pattern by the direct space method. The determined LT structural model was further optimized by DFT calculations. The DFT-relaxed LT structural model, together with the known RT structure, were used to fit the low-temperature XRD patterns. Figures 1c and S4b depict the Rietveld fit to the exemplary 138 K XRD pattern. The refined lattice parameters of the LT and the coexisting *RT* structures at 138 K are summarized in Table 1. The changes of the refined mass fractions, lattice parameters, and volume per formula unit (*V*/Z) of the LT and RT phases are shown in Figure 2 (note: the *a* of the LT structure is divided by 3 in order to directly compare with the *a* of the *RT* structure). The temperature range of the mass fraction change, i.e., 260 K–200 K, is consistent with the observed XRD peak intensity evolution. The mass fractions of LT and RT phases remain unchanged below 200 K, i.e., ∼60 mass % vs 40 mass %, indicating the end of the phase transition under the present measurement time duration and temperature window. The lattice parameters of both structures slightly contract with decreasing temperature. Interestingly, the volume per formula unit of the LT structure is noticeably smaller than that of the RT structure measured at the same low temperatures, indicating a denser packing of the ellipsoidal B_10_H_10_^2–^ cages in the LT structure. Particularly, the *a*/3 and *b* lattice parameters of the LT structure are smaller, while the *c* lattice parameter is larger than their corresponding lattice counterparts of the RT structure at the same temperature (Figure 2c).

**Table 1:** Refined Lattice Parameters of the Ordered Structures of K_2_B_10_H_10_ and KCB_9_H_10_

	K_2_B_10_H_10_				K-1-CB_9_H_10_		
T (K)	138	138	298	480	101	101	298
phase frac. (wt.%)	60.0(1)	40.0(1)	100	100	79.6(9)	20.4(9)	100
crystal system	monoclinic	monoclinic	monoclinic	monoclinic	orthorhombic	orthorhombic	tetragonal
space group	*P*2_1_/*n*	*P*2_1_/*n*	*P*2_1_/*n*	*P*2_1_/*n*	*C*222_1_	*I*222	*P*4̅2_1_/*m*
*a* (Å)	37.887(3)	12.766(1)	12.857(1)	13.027(1)	18.0149(5)	9.015(1)	9.2340(3)
*b* (Å)	10.991(1)	11.165(1)	11.1874(9)	11.311(1)	18.2590(5)	9.131(1)	9.2340
*c* (Å)	6.9055(7)	6.7797(8)	6.8279(5)	6.8764(6)	20.3146(7)	20.333(3)	10.1899(4)
β (°)	93.751(6)	92.43(1)	93.302(4)	94.130(4)	90	90	90
*V* (Å^3^)	2869.3(7)	965.5(3)	980.5(2)	1010.6(2)	6682.2(4)	1673.6(3)	868.86(9)
*Z*	12	4	4	4	32	8	4
*V/Z* (Å^3^)	239.1	241.4	245.1	252.7	208.8	209.2	217.2

**Figure 2:**
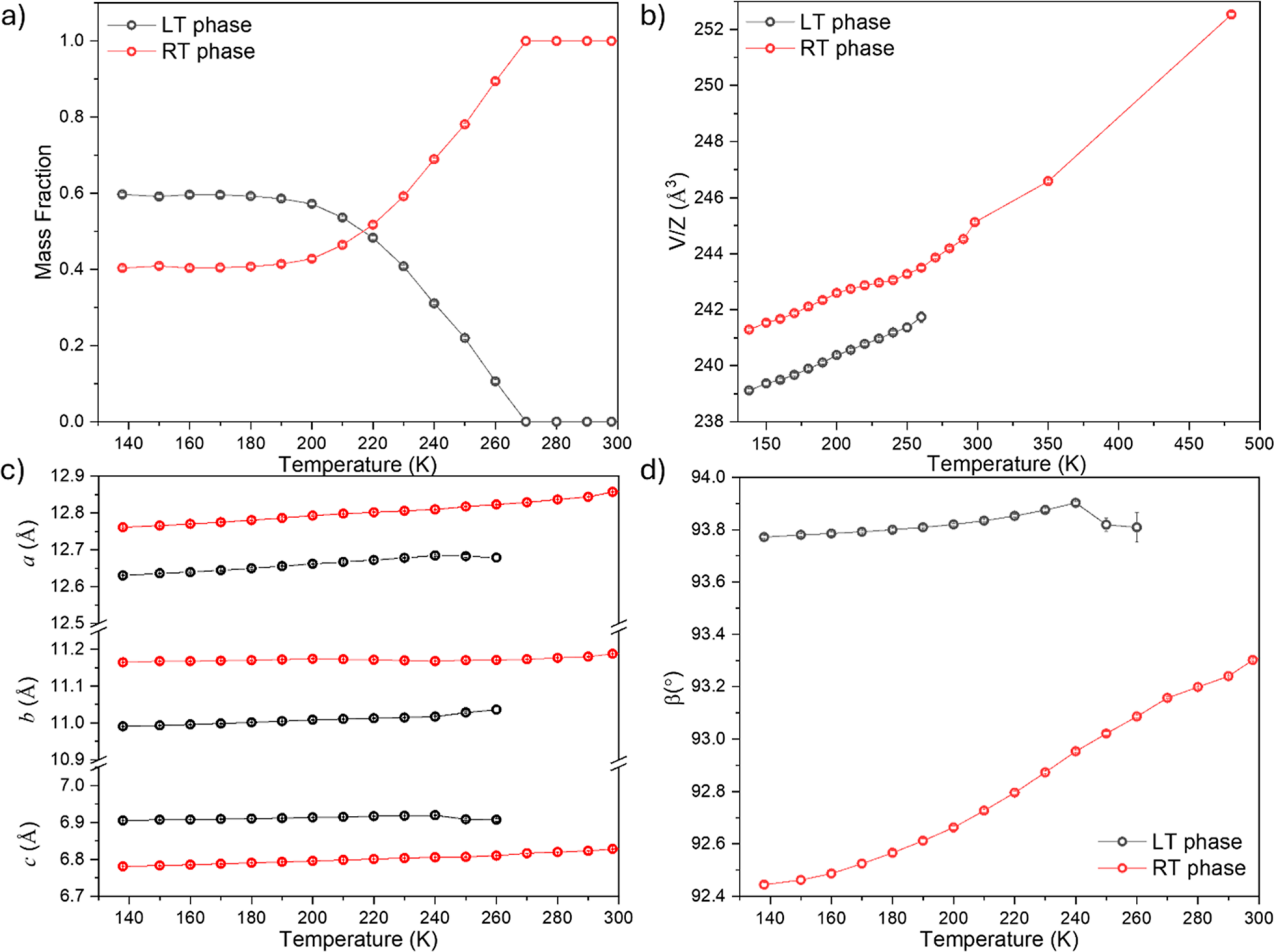
Changes of the crystallographic parameters of K_2_B_10_H_10_ RT (red symbols) and LT (black symbols) structures with temperature: (a) mass fraction; (b) volume per formula unit (*V/Z*); (c) lattice parameters (*a*, *b*, and *c*). The *a* lattice parameter of the LT structure has been divided by 3 to directly compare to that of the RT structure; and (d) β angle of the monoclinic structure. Note: the estimated standard deviations (e.g., numbers in the parentheses at 138 and 298 K listed in Table 1) shown in figures are too small to be visible in the figures.

Figures 3, S1 and S2 illustrate the crystal structure and anion packing of the LT phase and compare them with those of the RT structure. At a glance, the B_10_H_10_^2–^ anions in the LT structure have a similar, slightly distorted *hcp* scheme as that of the RT structure, e.g., each B_10_H_10_^2–^ is surrounded by 12 other nearest neighboring anions and directly coordinated to eight K^+^ cations, and each K^+^ is located in the distorted tetrahedral interstices. There is only one type of B_10_H_10_^2–^ anion in the RT structure, whereas there are three crystallographically independent B_10_H_10_^2–^ anions and six different K^+^ cations in one unit cell of the LT structure in the triple-size superlattice. As shown in Figure 3a, in the RT structure, the bicapped square antiprism of B_10_H_10_^2–^ anions all lie within the *ab* plane with their apical axes (the axes along the two apical B atoms as indicated by orange dashed lines) almost perpendicular to the ***c*** axis. A close inspection of the LT structure (Figure 3b) reveals that one of its three crystallographically independent B_10_H_10_^2–^ anions orientationally remains almost the same as those in the RT structure (orange dashed lines indicate the apical axes of the B_10_H_10_^2–^ anions). However, the other two types of anions significantly change their orientations; specifically, these two independent B_10_H_10_^2–^ anions tilt away from the *ab* plane to lie within the *ac* plane with their apical axes more aligned with the ***c*** axis. Therefore, the realignment of the apical axes of these B_10_H_10_^2–^ anions and the accommodation of their ellipsoidal shape would result in an expansion along *c* and contraction of the *a* and *b* lattices of a unit cell. Apparently, the transformation from the RT structure into the more complicated LT superstructure with a smaller *V/Z* must stem from the reorientation of the B_10_H_10_^2–^ anions. The overall K^+^ cation lattice basically maintains a similar arrangement in the LT structure to that in the RT structure. The overlays of these two structures are shown in Figure S6 for a direct comparison.

**Figure 3:**
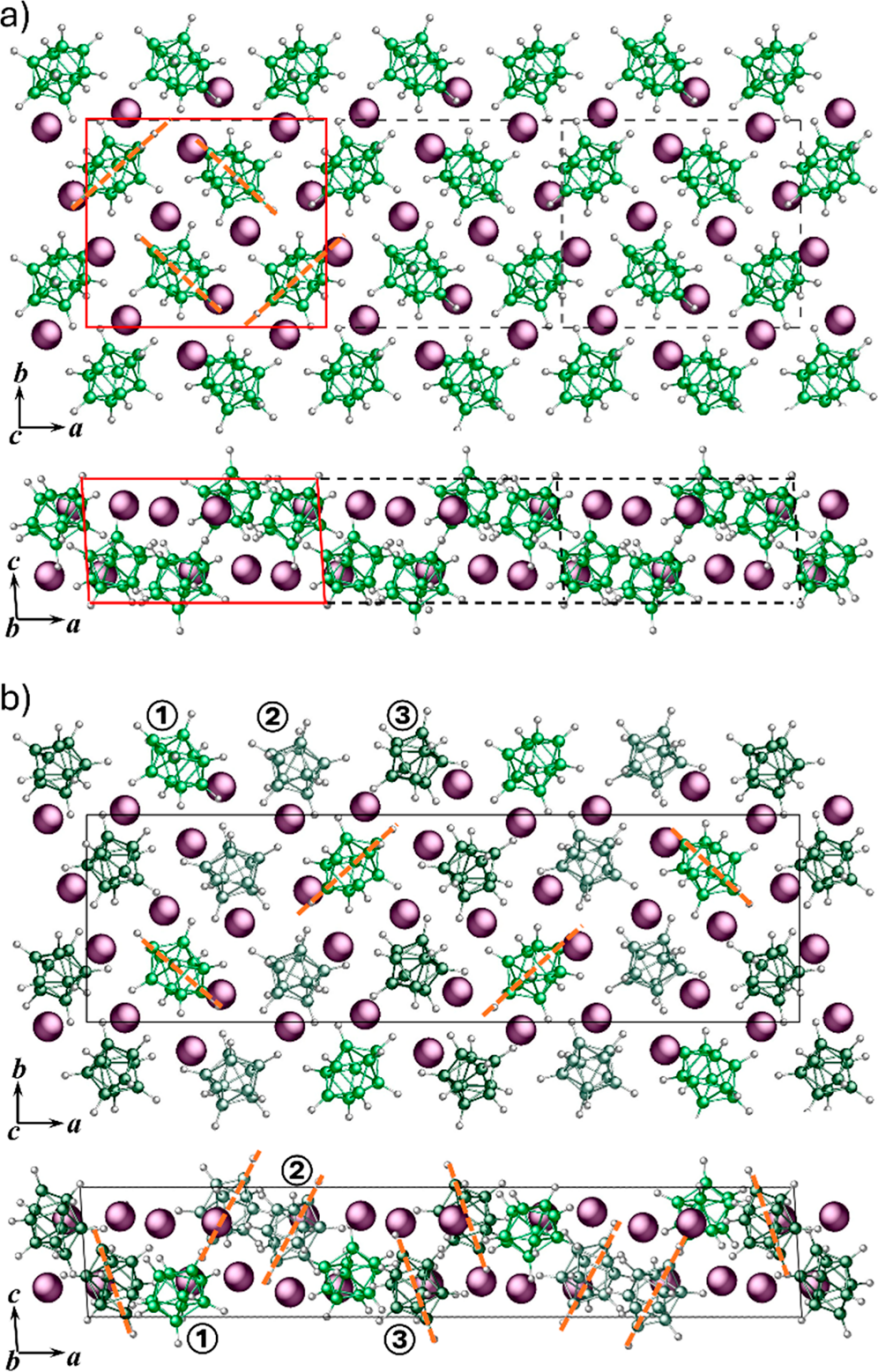
Comparison of the arrangement of K^+^ and B_10_H_10_^2–^ in the RT and LT structures of K_2_B_10_H_10_. (a) [001]- and [010]-views of the RT structure with its *a* lattice dimension tripled indicated by black dashed lines. Boron, hydrogen, and potassium atoms are shown by bright green, white, and purple spheres. Orange dashed lines indicate the apical axes of the B_10_H_10_^2–^ anions. Red solid lines highlight one unit cell of the RT structure. The apical axes of the bright green B_10_H_10_^2–^ anions in the RT structure all nearly lie within the *ab* plane (perpendicular to the **c** axis); (b) [001]- and [010]-views of the LT structure. *a*_LT_≈3*a*_RT_. Three crystallographically independent B_10_H_10_^2–^ anions in the LT structure are indicated by three different shades of green and labeled by numbers 1, 2, and 3. The apical axes of the bright green B_10_H_10_^2–^ anions labeled “1” are in similar orientations as those in the RT structure, while the apical axes of the other two types of anions labeled “2” and “3” nearly lie within the *ac* plane (tilting toward the ***c*** axis).

For the RT structure, the mean distances between B_10_H_10_^2–^ anion centers of mass are 7.081 Å and 7.043 Å at 298 and 138 K, respectively, due to the lattice contraction with temperature. However, at the same temperature of 138 K, the mean distance between B_10_H_10_^2–^ anion centers of mass in the LT structure is further reduced to 7.020 Å with the adjustment of the anion orientations. Therefore, the anion reorientation-induced phase transition facilitates a denser anion packing, consistent with the observed smaller volume of formula unit (*V/Z*) of the LT structure (Figure 2b). The anion orientation adjustment also fine-tunes the K positions as shown in the overlay comparison of the LT and RT structures (Figure S6), and results in more distorted tetrahedral interstitial sites for K^+^. Figure S7 displays K-anion coordination for the six crystallographically independent K^+^ cations in the LT structure; the two K tetrahedral interstices in the RT structure are also shown for comparison. The mean distances between cations and anion centers of mass range from 4.334 Å to 4.571 Å for the six K^+^ in the LT structure, whereas they are 4.398 Å and 4.537 Å for the two K^+^ in the RT structure.

### Crystal Structures of KCB_9_H_10_

The room-temperature diffraction pattern of KCB_9_H_10_ can be best fit by a tetragonal structural model with the space group *P*4̅2_1_ *m* (Figure 4a). In this structure (abbreviated as RT structure hereafter, Figure 5a), there is only one set of crystallographically independent CB_9_H_10_^–^ anions and K^+^ cations. The anions arrange themselves in a pseudo *fcc* packing scheme, and the K^+^ cations are at the octahedral interstice in the *fcc* anion sublattice but are clearly off-centered due to the repulsion from the more positively charged H­(C) of one CB_9_H_10_^–^ anion compared to the H­(B) sides of the other CB_9_H_10_^–^ anions.^5^ Consequently, there is a much longer distance between K^+^ and the mass center of that CB_9_H_10_^–^ anion (Figures 5d and S8a), e.g., 5.82 Å compared to the distances of 4.37Å–4.72 Å to the other five CB_9_H_10_^–^ anions, which are in orientations with their H­(C) atoms away from K^+^. Such a cation–anion arrangement to maximize the distance between H­(C) atoms and the surrounding cations is most energetically favorable and consistent with the other ACB_11_H_12_ (A = Li, Na, K, and Cs) compounds.^4,16,17,35^

**Figure 4:**
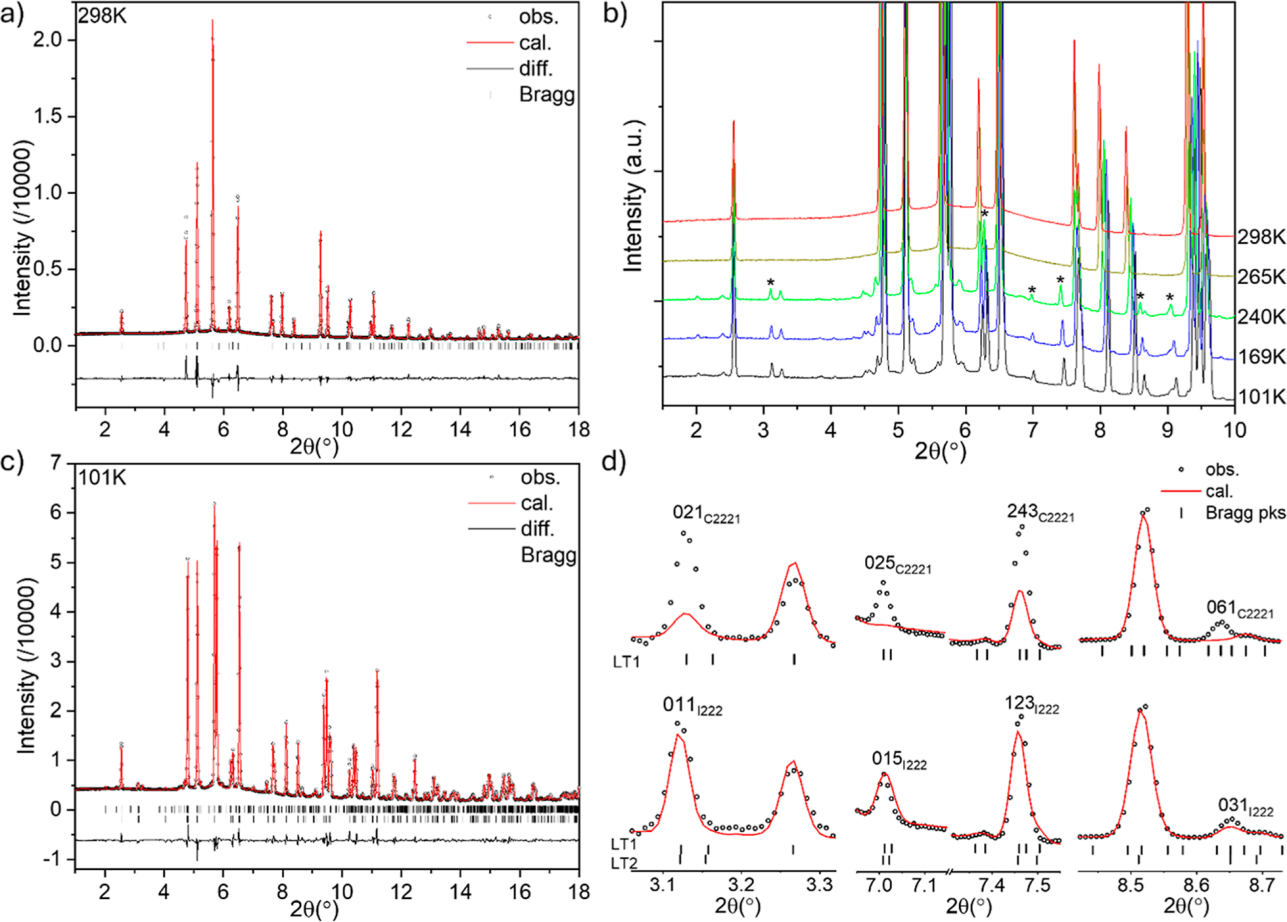
(a) Experimental (circles), fitted (line), and difference (line below observed and calculated patterns) synchrotron XRD profiles for KCB_9_H_10_ at 298 K. Vertical bars indicate the calculated positions of Bragg peaks. *R*_wp_ = 0.0859, *R*_p_ = 0.0555, χ^2^ = 2.47; (b) temperature-dependent XRD patterns indicating the phase transition from RT to LT structures with the appearance of satellite superlattice peaks from the LT1 phase. Peaks from LT2 phase are indicated by asterisks; (c) experimental (circles), fitted (line), and difference (line below observed and calculated patterns) synchrotron XRD profiles for KCB_9_H_10_ at 101 K. Vertical bars indicate the calculated positions of Bragg peaks of the LT1 phase (79.6(9) wt.%, top) and LT2 phase structures (20.4(9) wt.%, bottom). *R*_wp_ = 0.0521, *R*_p_ = 0.0383, and χ^2^ = 3.22; and (d) Rietveld fitting of representative diffraction peaks at 101 K using a sole LT1 model (top) and using LT1+LT2 two-phase models (bottom). XRD measurement wavelength λ = 0.45415 Å.

**Figure 5:**
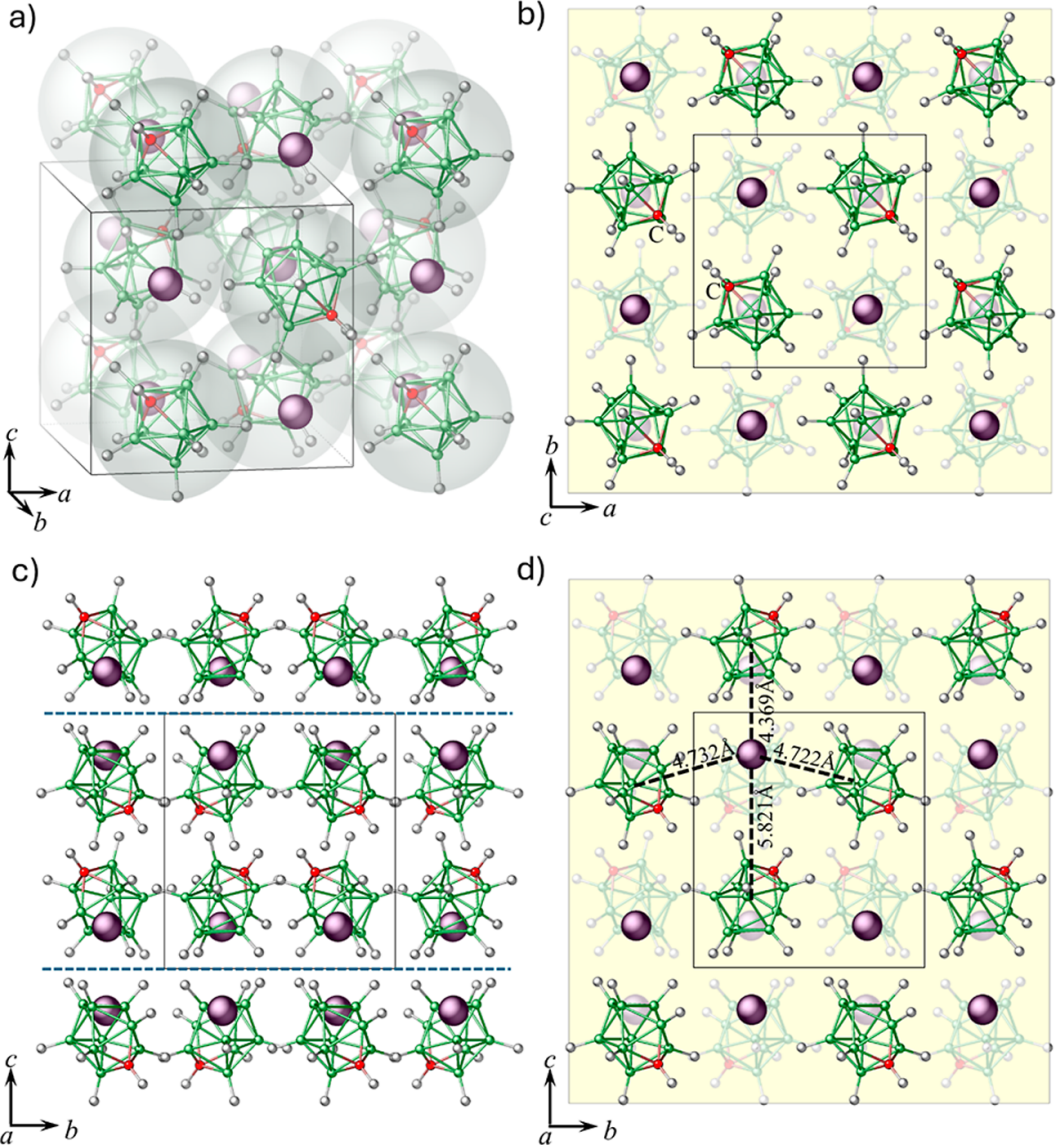
(a) Off-[010] view of the tetragonal RT structure of KCB_9_H_10_. Boron, carbon, hydrogen, and potassium atoms are shown by bright green, bright red, white, and purple solid spheres. The pseudo-*fcc* packing array of CB_9_H_10_^–^ anions is illustrated by large transparent gray spheres. Gray lines outline one unit cell; (b) [001]-view of the RT structure with one slice of CB_9_H_10_^–^ anions shown above a (00*l*) cutting plane and an adjacent slice of anions right beneath this plane shown in faded color. The red carbon atoms are also labeled with “C”. The (00*l*) plane is indicated by a light-yellow rectangle. Gray lines inside the light-yellow (00*l*) plane outline the edges of a unit cell; (c) [100]-view of the RT structure with C–H apexes of the CB_9_H_10_^–^ anions in the two neighboring *ab*-planes tilting inward, forming the “H­(C)-sandwiched” anion-layer pairs. Blue dashed lines highlight one such pair of layers. K^+^ cations locate between these pairs of anion layers and stay away from the H­(C) atoms; and (d) [100]-view of a front slice of CB_9_H_10_^–^ anions from (c). The (*h*00) cutting plane is indicated by a light-yellow rectangle. An adjacent slice of anions right behind this plane is also shown in faded color. Gray lines inside the light-yellow (*h*00) cutting plane outline one unit cell. The off-centered K^+^ octahedral coordination is illustrated with a longer distance from one CB_9_H_10_^–^ anion, whose H­(C) is pointing to the K^+^ cation.

Viewed in the **c** direction (e.g., Figure 5b [001]-view), the H­(C) atoms of CB_9_H_10_^–^ anions within the same *ab*-plane are aiming toward each other in pairs (i.e., a face-to-face orientation), which would aggravate the repulsion between anions in the *ab*-planes. Viewed perpendicular to the **c** direction (e.g., Figure 5c,d [100]-view), the C–H apexes of the CB_9_H_10_^–^ anions in the two adjacent *ab* planes tilt inward within this pair of anion layers, while their respective B–H apexes face outward. This structural arrangement aligns the carbon-bound hydrogen atoms within the interior interface of the bilayer, thereby forming what we define as “H­(C)-sandwiched” anion-layer pairs. It should be noted that the apical axes of these anions between the adjacent *ab* planes are nearly perpendicular to each other, which prevents their positive H­(C) atoms from directly confronting each other in the “H­(C)-sandwiched” anion-layer pairs (Figure 5d). Therefore, the inward tilting of the C–H apexes would not cause large unfavorable repulsive interactions in these coupled anion layers along the **c** axis. Because of the strong electrostatic repulsion between the K^+^ cations and the highly positive H­(C) vertices, K^+^ cations all concentrate in the space between the pairs of such “H­(C)-sandwiched” anion layers to maximize their distance from these unfavorable H­(C) contacts, in accordance with the off-centered octahedral coordination of K^+^ due to the repulsion from the H­(C) vertex of one CB_9_H_10_ anion in a K^+^ coordination sphere (Figure 5d).

Upon cooling below 250 K, many satellite superlattice peaks appear and persist throughout the measured low-temperature range down to 100 K (Figure 4b). All the peaks in the low-temperature XRD patterns below 150 K can be indexed into an orthorhombic unit cell with approximately twice the *a*, *b*, and *c* lattices of the RT structure (i.e., ∼2*a*_RT_ × 2*b*_RT_ × 2*c*_RT_) in the space group *C*222_1_ (abbreviated as LT1 structure). The LT1 crystal structure was then determined using this large cell with four independent CB_9_H_10_^–^ anions and four independent K^+^ cations. Intriguingly, the intensities of several peaks cannot be satisfactorily fitted using the LT1 structure model alone, although the remaining satellite superlattice peaks can be reasonably well fitted (Figure 4d). The careful inspection of the temperature-dependent XRD patterns also indicates that the intensities of these peaks (asterisked peaks in Figure 4b) seem to increase slightly with decreasing temperature while the intensities of the other satellite peaks remain almost unchanged. Considering this special set of superlattice peaks together with the main strong peaks, we could derive another low-temperature structure with a smaller orthorhombic unit cell in space group *I*222 (abbreviated as LT2 structure). This structure has twice the *c* lattice and similar *a* and *b* lattice parameters compared to the RT structure (i.e., ∼*a*_RT_ × *b*_RT_ × 2*c*_RT_). Rietveld refinement using the LT1+LT2 two-phase models results in a satisfactory fit of the low-temperature XRD patterns (Figure 4c,d). Apparently, LT1 and LT2 appear concurrently at the transition onset and coexist below 250 K. The refined crystallographic parameters are summarized in Table 1, and the temperature-dependent changes of the mass fractions, lattice parameters, and the volume per formula unit (*V*/Z) of all phases are shown in Figure 6. Different from K_2_B_10_H_10_, where the mass fractions of RT and LT monoclinic phases show a gradual change with temperature, the phase transition in KCB_9_H_10_ is a relatively abrupt tetragonal-to-orthorhombic (*t*-to-*o*) transition, consistent with the sudden appearance of XRD superlattice peaks upon cooling. The mass fractions of the LT1 and LT2 phases slightly change from 82.0(9) wt.%/18.0(9) wt.% to 79.6(9) wt.%/20.4(9) wt.% from 240 to 100 K, respectively, consistent with the observed slight increase in peak intensities of the LT2 structure with decreasing temperature (Figure 4b). The lattice parameters of all structures contract with decreasing temperature, while the comparison of the normalized lattice parameters suggests that the *t*-to-*o* phase transition would lead to a small expansion in *c*, and contractions in *a* and *b* lattice to different extents.

**Figure 6:**
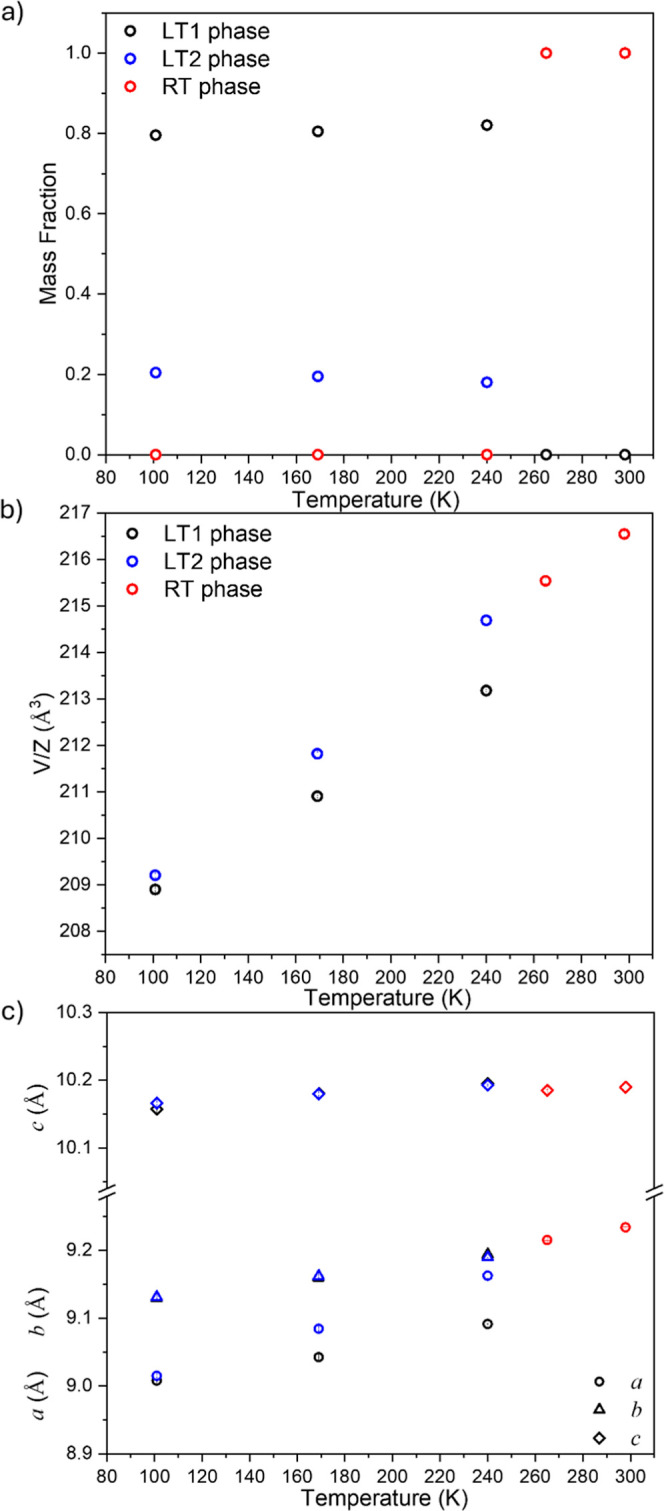
Changes of the crystallographic parameters of KCB_9_H_10_ RT (red symbols), LT1 (black symbols), and LT2 (blue symbols) structures with temperature: (a) mass fraction; (b) volume per formula unit (*V/Z*); and (c) lattice parameters (*a*, *b*, and *c*). The *a*, *b*, and *c* lattice parameters of the LT1 structure and the *c* lattice parameter of the LT2 structure have been divided by 2 to directly compare to those of the RT structure. Note: the estimated standard deviations shown in figures are too small to be visible.

Since LT1 is the dominant phase (∼80 wt.%) in the low-temperature range, a solid refinement of the minor phase LT2 cannot be attained with sufficient precision from the present powder XRD data. We will therefore only focus on the refined LT1 crystal structure, while the DFT-calculated structure model of the LT2 phase is presented in the Supporting Information. As shown in Figure 7, the *t*-to-*o* transition generates four crystallographically independent CB_9_H_10_^–^ anions and four independent K^+^ cations in the LT1 structure. The overall anion packing in the LT1 structure remains a pseudo *fcc* type with K^+^ cations in the octahedral interstices (Figure 7a). Compared to the orientations of the single type of CB_9_H_10_^–^ anions in the RT structure, the four crystallographically different anions in the LT1 structure create extra sets of inward- and outward-plane orientations of their apical axes, i.e., negative and positive **b** directions alternating along the **a** axis, and negative and positive **a** directions alternating along the **b** axis (Figure 7b,c). These CB_9_H_10_^–^ anions in the adjacent *ab* planes also align into “H­(C)-sandwiched” anion-layer pairs with their C–H vertices facing inward within each pair of layers.

**Figure 7:**
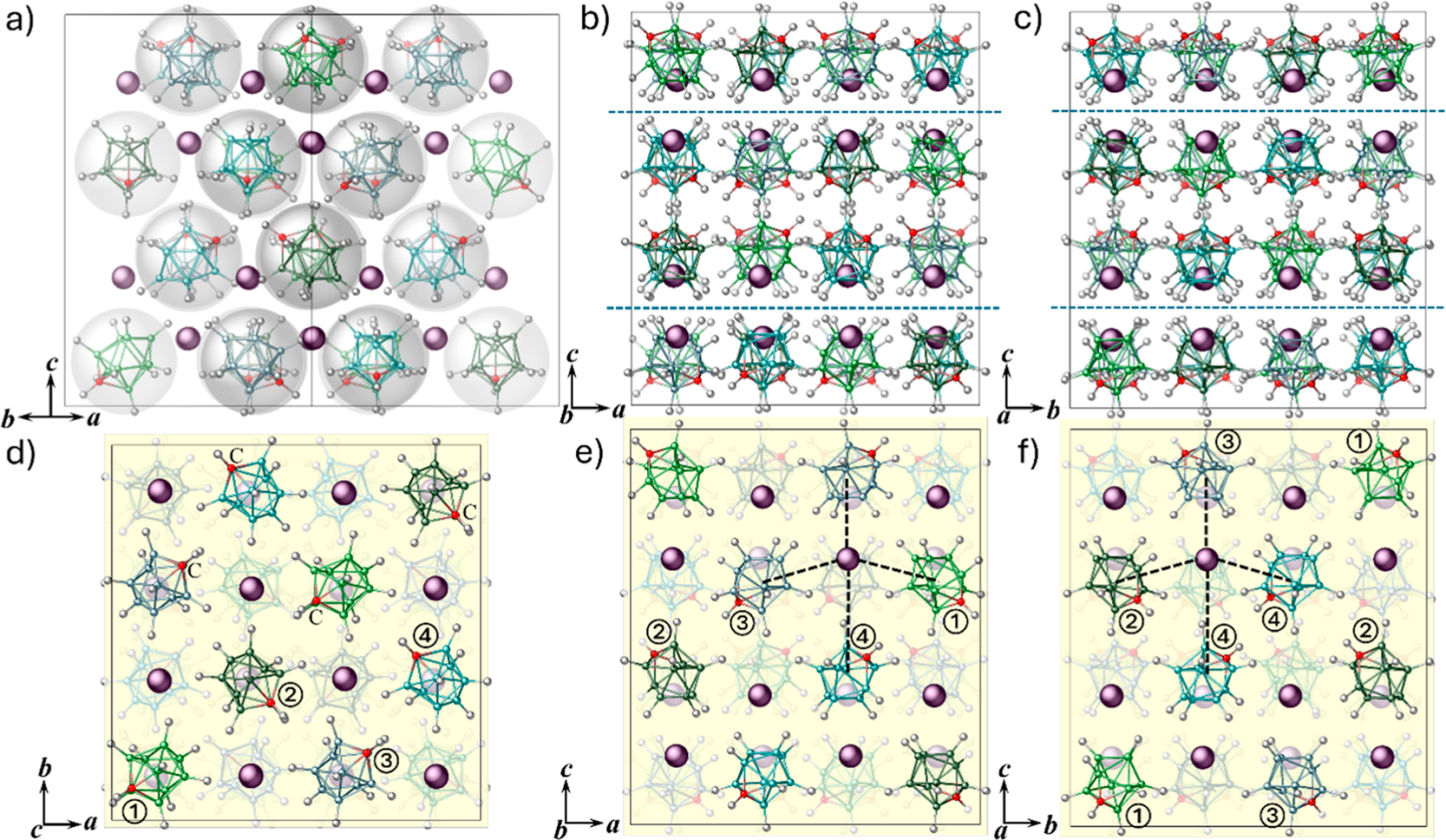
(a) [110] view of KCB_9_H_10_ orthorhombic LT1 structure. *a*_LT1_ × *b*_LT1_ × *c*_LT1_ ≈ 2*a*_RT_ × 2*b*_RT_ × 2*c*_RT_. Carbon, hydrogen, and potassium atoms are shown by bright red, white, and purple solid spheres. Four crystallographically independent CB_9_H_10_^–^ anions in the LT1 structure are indicated by bright green, dark green, light blue, and cyan, and labeled by numbers 1, 2, 3, and 4 in (d–f). The red carbon atoms are also labeled with “C” in (d). The pseudo-*fcc* packing array of CB_9_H_10_^–^ anions is illustrated by large transparent gray spheres. Gray lines outline a unit cell; (b) [010]- and (c) [100] views of KCB_9_H_10_ LT1 structure with C–H apexes of the CB_9_H_10_^–^ anions in the two neighboring *ab*-planes tilting inward, forming the “H­(C)-sandwiched” anion-layer pairs. Blue dashed lines highlight one such pair of layers. K^+^ cations shown by purple spheres locate between pairs of the anion layers and away from the H­(C) atoms. Gray lines outline a unit cell; (d) [001]-view of the LT1 structure with one slice of CB_9_H_10_^–^ anions shown above a (00*l*) cutting plane and an adjacent slice of anions right beneath this plane shown in faded color. The (00*l*) cutting plane is indicated by a light-yellow rectangle. Gray lines inside the light-yellow (00*l*) cutting plane outline the edges of one unit cell; (e) [010] view of a front slice of CB_9_H_10_^–^ anions from (b). The (0*k*0) cutting plane is indicated by a light-yellow rectangle. An adjacent slice of anions right behind this plane is also shown in faded color. The off-centered K^+^ coordination is illustrated on one of the four crystallographically independent K^+^ cations. The distances between K^+^ cations and the mass centers of CB_9_H_10_^–^ anions are shown in Figure S8; (f) [100]-view of a front slice of CB_9_H_10_^–^ anions from (c). The (*h*00) cutting plane is indicated in a light-yellow rectangle. An adjacent slice of anions right behind this plane is also shown in faded color. The H­(C) atoms of two neighboring CB_9_H_10_^–^ anions (both in an inward-plane orientation labeled by “4”) are aiming toward each other.

Compared to the RT structure, where the single type of H­(C) atoms are not directly pointing to each other between the two layers in an “H­(C)-sandwiched” anion-layer pair (Figure 5d), the “directional” anion apical axes in the LT1 structure can generate different propagating patterns of the H­(C)-pointing directions along the **a** and **b** axes within the “H­(C)-sandwiched” anion-layer pairs. When spreading along the **a** direction (Figure 7e), the H­(C) atoms within an “H­(C)-sandwiched” anion-layer pair can avoid directly pointing toward each other through perpendicular (e.g., anions labeled “2” and “3”), antiparallel (e.g., anions labeled “3” and “4”) and inward- and outward-plane (e.g., anions labeled “1” and “2”) apical axial orientations. While spreading along the **b** direction (Figure 7f), some anions within an “H­(C)-sandwiched” anion-layer pair can also avert direct interaction between their H­(C) atoms through perpendicular (e.g., anions labeled “2” and “4”) and antiparallel (e.g., two anions labeled “2” in two adjacent unit cells) orientations. However, other anions could generate a “local” anion pair with their H­(C) atoms aiming toward each other (i.e., face-to-face orientation), such as the two neighboring anions labeled “4” in the present unit cell that both have an inward-plane orientation. Such face-to-face anion-pairs would presumably increase the repulsive interaction between the two layers in an “H­(C)-sandwiched” anion-layer pair and expand the *c* lattice, but to a limited extent because this interaction is less dense and only occurs partially in the LT1 structure compared to the prevalent face-to-face pairings observed throughout the RT structure. This is consistent with the slight expansion observed in the *c* lattice of the LT1 structure (Figure 6c). Due to the specific apical orientation of these anions, i.e., nearly along <011> directions as indicated in Figure 7f, the partial presence of face-to-face anion-pairs would also cause an expansion in the *b* lattice, whose effect will be discussed below. On the other hand, when viewed in the **c** direction (e.g., [001]-view in Figure 7d), the four crystallographically different CB_9_H_10_^–^ anions within each individual anion-layer in the *ab* planes are able to orientate in such a way that the apical axis of each anion is almost perpendicular to those of its surrounding anion neighbors. This anion arrangement largely alleviates the repulsive interaction between the confronting face-to-face H­(C) atoms of the neighboring anions in the *ab*-plane of the RT structure (Figure 5b). Such a closely knitted in-plane arrangement of anions in the LT1 structure would effectively shorten the distances between anions in the *ab*-planes, resulting in a contraction in the *a* and *b* lattice parameters. Because of the presence of the forementioned face-to-face anion-pairs propagating along the **b** direction, the contraction in the *b* lattice would be offset and less significant than that in the *a* lattice, in accord with the observed trend of lattice changes in Figure 6c.

Likewise, K^+^ cations in the LT1 structure all situate at the B–H sides of the “H­(C)-sandwiched” CB_9_H_10_^–^ layer pairs, staying away from the H­(C) atoms of the CB_9_H_10_^–^ anions (Figure 7b,c). Each K^+^ in the LT1 structure is also located in an off-centered octahedral coordination environment, with the distances of the cation to the mass center of the anions in the range of 4.15 Å–5.99 Å (Figure S8b). The distances of K^+^ to the mass centers of the farthest CB_9_H_10_^–^ anions are longer than that of the RT structure (5.87 Å–5.99 Å compared to 5.82 Å), while the distances to the mass centers of the other five CB_9_H_10_^–^ anions are generally shorter than those in the RT structure, resulting in a shorter mean distance of cation–anion mass center (4.81 Å in LT1 vs 4.85 Å in RT). Apparently, the changes in the anion–cation distances resulted from the LT1 structural variant discussed above could help to further reduce the repulsive forces from the highly positive H­(C) of the CB_9_H_10_^–^ ^5^ and to promote a more effective bonding between K^+^ and the surrounding anions.

The additional directional component to the apical axis of the ellipsoidal polarized CB_9_H_10_^–^ anion can generate extra sets of inward/outward-plane canting of the ellipsoidal anions besides the simple anion orientation adjustment. Such structural variability provides more flexibility to lessen the unfavorable repulsive forces between the neighboring CB_9_H_10_^–^ anions, which may lead to various structural polymorphs with different improved packing efficiencies and cohesive energies. LT1 represents one type of structural variant resulting from concerted reorientation of anions to lessen the repulsion between anions and to enhance the cation–anion bonding. One of the plausible structural arrangements of the minor ordered phase observed in the low-temperature diffraction patterns (Figure 4) might be the orthorhombic LT2 model. However, because a rigorous experimental refinement cannot be independently conducted on this component, the LT2 structure must be considered only a tentative, DFT-calculated structural model. In this representative LT2 model, there is only one crystallographically independent K^+^ cation site and one independent CB_9_H_10_^–^ anion site. Both are in the general (*x*, *y*, *z*) positions. We will not discuss the structural details of this model further but only illustrate it in Figure S9.

The neutron vibrational spectra of K_2_B_10_H_10_ and KCB_9_H_10_ at 4 K are shown in Figures 8 and 9, respectively, and compared with the simulated phonon spectra of the DFT-optimized low-temperature structures as well as the isolated B_10_H_10_^2–^ (Figure 8) and CB_9_H_10_^–^ anions (Figure 9). For K_2_B_10_H_10_, the simulated spectrum is a 60:40 average of the LT and RT structures; for KCB_9_H_10_, the simulated spectrum is an 80:20 average of the LT1 and LT2 structures. These weighted averages match the mass phase ratios determined at the lowest temperatures by XRD. It should be noted that the LT and RT K_2_B_10_H_10_ monoclinic structures exhibit comparatively similar PDOSs (see Figure S10) as do the LT1 and LT2 KCB_9_H_10_ orthorhombic structures (see Figure S11), so any differences due to differences in spectral averaging are subtle at best. The importance of the particular crystal structure arrangements between cations and anions is more obvious when comparing these simulated spectra with those for the isolated anions where larger PDOS deviations are observed. As the vibrational features in this measured energy range mainly involve deformation modes of the anions, it is evident from the spectral comparison of the isolated and lattice-confined anions that those particular anion deformation modes with energies above ∼90–100 meV are noticeably perturbed by the presence of the surrounding K^+^ cation matrix. It has been shown previously that the PDOSs for *closo*-borates are indeed sensitive to the crystal structure details.^4,22,23,26,36^ The overall good agreement between the experimental and calculated PDOSs for both compounds further corroborates the low-temperature structures of K_2_B_12_H_10_ and KCB_9_H_10_ determined by X-ray powder diffraction data.

**Figure 8:**
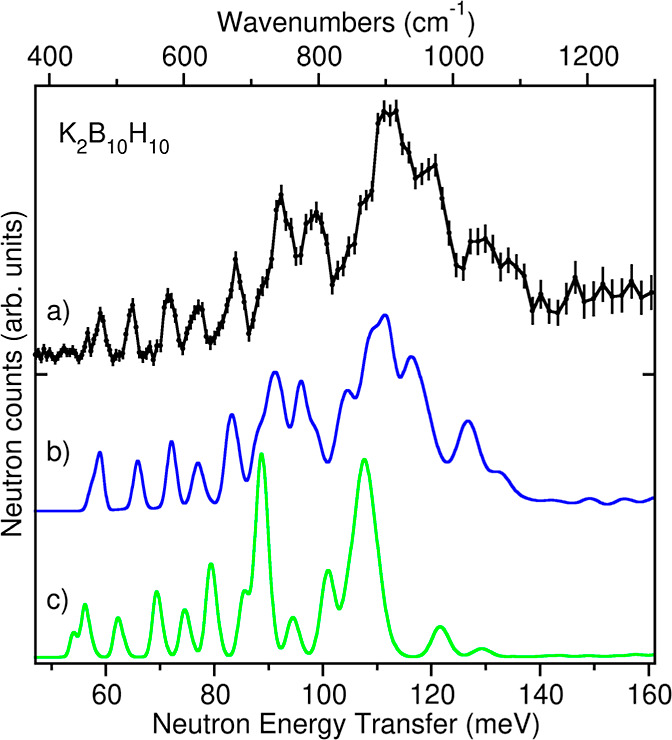
(a) Neutron vibrational spectrum of K_2_B_10_H_10_ at 4 K compared with (b) the DFT-simulated one + two-phonon density of states representing the 60:40 weighted average of the respective LT and RT monoclinic structures and (c) the isolated B_10_H_10_^2–^ anion spectrum. (*N*.*B*., 1 meV ≈ 8.066 cm^–1^).

**Figure 9:**
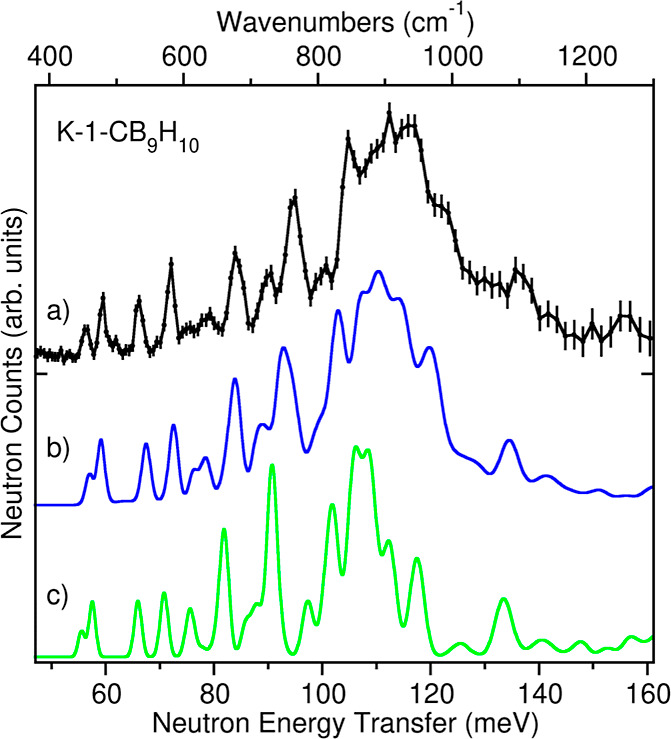
(a) Neutron vibrational spectrum of K-1-CB_9_H_10_ at 4 K compared with (b) the DFT-simulated one + two-phonon density of states representing the 80:20 weighted average of the respective orthorhombic LT1 and LT2 spectra and (c) the isolated 1-CB_9_H_10_^–^ anion spectrum. (*N*.*B*., 1 meV ≈ 8.066 cm^–1^.).

The gradual, prolonged phase transition and the RT/LT phase coexistence observed in the in situ XRD results for K_2_B_10_H_10_ as well as the concurrent emergence and coexistence of the dual LT1/LT2 phases following the rapid first-order transition in KCB_9_H_10_ warrant further thermodynamic consideration. The different phase evolution behaviors of K_2_B_10_H_10_ and KCB_9_H_10_ observed in the temperature-dependent XRD experiments are supported by the DSC measurements. As depicted in Figure 10, K_2_B_10_H_10_ does not exhibit a noticeable phase transition signal below room temperature, consistent with the gradual continuous phase transition observed in XRD results (Figures 1 and 2), whereas KCB_9_H_10_ shows a clear reversible first-order transition reflected by a sharp enthalpic peak centered near 254 K (during the heating segment), in accordance with the sudden appearance of the superlattice peaks from the LT1 and LT2 phases and rapid phase fraction changes observed in its XRD results (Figures 4 and 6). To quantify the underlying driving forces governing these transitions, Helmholtz free energies (F) were calculated for the respective RT and LT structures of both compounds.

**Figure 10:**
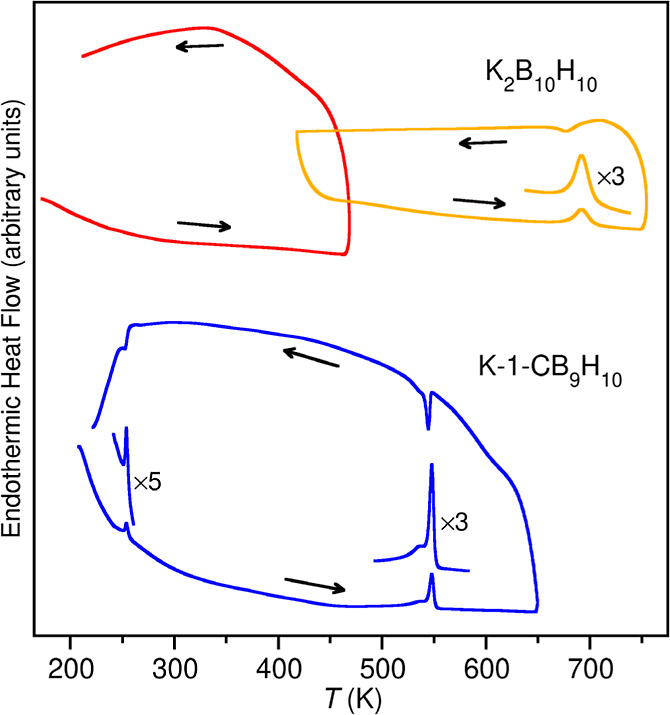
DSC scans for K_2_B_10_H_10_ and KCB_9_H_10_ in heating and cooling at ± 20 K min^–1^. Endothermic signals are normalized by sample masses. For K_2_B_10_H_10_, the wider temperature range was explored by two separate overlapping heating/cooling cycling programs as indicated by the red scan spanning from 200 to 470 K and the orange scan spanning from 420 to 750 K. Arrows indicate the temperature direction during scans.

For K_2_B_10_H_10_, at 0 K, the calculated Helmholtz free energy of the LT structure is lower than that of the RT phase by a mere ∼1.5 kJ/mol. This marginal energetic differentiation points to a fairly flat or shallow potential energy landscape separating these two ordered configurations. Conversely, at 298 K, the RT phase achieves thermodynamic stability over the LT phase by ∼5.7 kJ/mol, establishing that the RT structure is robustly stabilized by entropic contributions. According to the ideal Landau theory,^37,38^ macroscopic phase coexistence is strictly forbidden in an ideal, equilibrium continuous (second-order) transition. In a real-world powder sample, however, the exceptionally shallow landscape at 0 K renders the RT-to-LT transformation highly susceptible to nonequilibrium effects rather than allowing for an ideal thermodynamic equilibrium state. Given that our experimental data revealed a broad phase coexistence window in the in situ XRD patterns but no detectable latent heat in the DSC measurements, the phase transition in K_2_B_10_H_10_ cannot be definitively classified as purely continuous or first-order. In a continuous transition model, the structural rearrangement between the RT and LT configurations requires a long-range, highly cooperative reorientation of the large bulky elliptical divalent B_10_H_10_^2–^ anions. Because the thermal energy available to surmount the activation barriers for such large-scale reorientations drops sharply at low temperatures, the system encounters rising kinetic friction. This kinetic limitation can effectively interrupt the continuous transformation, conceivably trapping a portion of the RT phase alongside the emerging LT phase.^39,40^ Alternatively, as detailed in the literature,^41,42^ many solid-state structural transitions that appear second-order are in fact weakly first-order. The absence of a macroscopically observable latent heat in calorimetry often stems from the practical resolution limits when measuring exceptionally small enthalpy changes within standard laboratory time scales. If this is the case for K_2_B_10_H_10_, the enthalpy change associated with the discontinuous structural shift between the two ordered orientations would be small enough to elude DSC detection, mimicking a continuous transition. Furthermore, a weakly first-order transition operating with an exceptionally weak thermodynamic driving force (Δ*F* ∼ 1.5 kJ/mol) will heavily suppress the nucleation and growth rates of the emerging phase.^43^ Lacking the thermodynamic driving potential required to quickly and completely convert the lattice from one discrete orientation to another, the system exhibits long-lived, mixed-phase signatures in the XRD data. Therefore, the observed broad mixed-phase window in K_2_B_10_H_10_ is best understood as an extrinsic, nonequilibrium phenomenon rather than a stable thermodynamic state. The interplay of a nearly flat energy landscape, severe kinetic friction associated with low-temperature anion reorientation, and slow nucleation/growth kinetics from a potentially weak first-order transition all combine to prevent the system from fully equilibrating. Under the rapid data-collection time scales of the in situ synchrotron experiment, these factors effectively freeze a portion of the ordered RT phase in a metastable state alongside the emerging ordered LT phase.

For KCB_9_H_10_, at 0 K, the calculated Helmholtz free energy (F) of the LT1 structure is slightly lower than that of the LT2 structure by only 0.8 kJ/mol. Because this value falls below the standard DFT calculations error bar (∼1 kJ/mol), these two structures are practically degenerate in energy. This energetic degeneracy directly explains their thermodynamic coexistence observed experimentally. At 0 K, the free energy of the RT structure is higher that of the LT2 structure by ∼5.3 kJ/mol, indicating that the RT structure is indeed thermodynamically unstable at low temperatures. Conversely, at 298 K, entropy contributions reverse this stability order, rendering the RT structure more stable than the LT1 and LT2 structures, by ∼7.4 and 8.2 kJ/mol, respectively. The substantial Δ*F* between the RT and the LT1/LT2 structures provides a sufficient thermodynamic driving force to facilitate a sharp first-order phase transition and a complete conversion from the RT phase. Due to the nearly degenerate free energies of the LT1 and LT2 structures, these two low-temperature phases emerge concurrently at the transition onset and continue to coexist following the transformation from the RT phase upon cooling. Our in situ XRD data reveal a subtle, temperature-dependent growth in the phase fraction of LT2 upon cooling (e.g., from 18.0(9) wt.% at 240 K to 20.4(9) wt.% at 100 K accompanied by a corresponding increase in the LT2 Bragg peak intensities, as shown in Figure 4b). This behavior could stem from differences in their respective crystallization kinetics during the in situ synchrotron measurement. While it is theoretically possible that given infinite time the system might evolve toward a single thermodynamically stable phase, the energetic degeneracy of the LT1 and LT2 structures, combined with the exceptionally minor variations observed across this wide temperature range, suggests that a complete phase transformation is unlikely under practical experimental conditions.

## Discussion

For all alkali-metal dodecahydro-*closo*-dodecaborate (A_2_B_12_H_12_, A = Li, Na, K, Rb, and Cs) with the quasi-spherical B_12_H_12_^2–^ anions, the ordered structure of each compound can be maintained without any phase transition in their corresponding ordered-structure temperature range.^12,15,19^ Unlike the dodecaborates (A_2_B_12_H_12_), for alkali-metal decahydro-*closo*-decaborates (A_2_B_10_H_10_), the quasi-ellipsoidal B_10_H_10_^2–^ anions lead to varied ordered-phase behaviors depending on the alkali cation type. Both the orientations and the packing scheme of B_10_H_10_^2–^ anions can remain unchanged in the ordered structures of Li_2_B_10_H_10_ and Na_2_B_10_H_10_ from room temperature to low temperature.^22,23^ While there is a monoclinic-to-triclinic phase transition in Rb_2_B_10_H_10_, the anion orientation and overall packing in these two phases are approximately retained with just lattice deformation.^24^ Therefore, it is surprising to observe a distinct phase transition from the RT-ordered to the LT-ordered structure in K_2_B_10_H_10_ induced by the anion reorientation, even though the overall packing pattern of the B_10_H_10_^2–^ anions can still be sustained throughout the studied temperature range.

To understand this abnormality, it would be useful to consider the Goldschmidt geometric packing criteria^44,45^ directed by the cation/anion radius ratio. The effective radius of the polyborate anion was empirically derived by subtracting the Shannon ionic radius^46^ of the corresponding alkali-metal cation from the average distance between the cations and the anion centers of mass. For the reported structures of Li_2_B_10_H_10_^22^ and Na_2_B_10_H_10_,^23^ the respective radius ratios (*r*_A+_/*r*_anion_) are 0.164 and 0.307, which favor triangle and tetrahedral arrangements of anions around a cation according to the geometric packing criteria and are indeed consistent with the observed cation coordination in the corresponding compounds. It should also be mentioned that the geometry packing criteria dictate the maximum coordination number, and lower coordination numbers are necessary to account for the stoichiometry of the compound. For instance, the *r*_Rb+_/*r*_anion_ ratio of Rb_2_B_10_H_10_ suggests that the maximum coordination number of each ion can be 6 based on only the relative size. Since there are twice as many cations as anions in Rb_2_B_10_H_10_, its 2:1 stoichiometry suggests that the cation coordination number should be half that of the anions. Indeed, the Rb^+^ cations were observed to be in the tetrahedral interstices in both its RT and LT structures, which differ slightly in lattice distortion.

In the RT structure of K_2_B_10_H_10_, K^+^ cations situate in the distorted tetrahedral interstices surrounded by four B_10_H_10_^2–^ anions and the radius ratio *r*_K+_/*r*_anion_ is 0.445, which is out of the range favoring a tetrahedral 4-fold coordination (0.225–0.414) but within the range of the 6-fold coordination criteria (0.414–0.732). Apparently, the 2:1 stoichiometry of cations and anions in K_2_B_10_H_10_ also needs to be accounted for to rationalize the tetrahedral cation coordination geometry. In addition, if we assume that the K^+^ cations remain in contact with the anions with a *r*_K+_/*r*_anion_ exceeding the cation tetrahedral packing criteria, then the B_10_H_10_^2–^ anions would be pushed apart in order to reduce the anion–anion repulsion and form a more stable structure. Indeed, instead of the mutual contact between anion spheres in an ideal close-packing scheme, we observed a pseudo-*hcp* anion arrangement (with 12 nearest neighbors with the distances between their mass centers in the range of 6.64 Å–7.72 Å) in K_2_B_10_H_10_ with relatively loosely packed anions (Figure S1). The arrangement of these anions in K_2_B_10_H_10_ can even be approximated as a severely distorted *bcc* packing (with 14 nearest neighbors, and less dense than *hcp*) if two more next nearest anion neighbors with their distance to the center anion as 8.78 Å are also included (Figure S12). Besides, compared to the *fcc* close packing (or *hcp*) of the almost spherical B_12_H_12_^2–^, the putative random packing of the congruent ellipsoidal B_10_H_10_^2–^ anions would be less dense.^47^ Such combined effects would therefore provide more leeway to accommodate the ellipsoidal B_10_H_10_^–^-anions in order to improve the packing efficiency and cohesive energy, as manifested by the adjustment of the orientations of some anions in K_2_B_10_H_10_ and the resulting deformed unit cell to accommodate the packing upon cooling.

Consistent with structural changes observed from the known *closo*-borates and their *closo*-carborate counterparts, the changes in both the anion valence and polarization from B_10_H_10_^2–^ to CB_9_H_10_^–^ directly lead to changes in stoichiometry and cation–anion interactions, which will certainly render the formation of different structures and the consequentially desired properties such as ionic conductivity. When the stoichiometry is changed from 2:1 (K_2_B_10_H_10_) to 1:1 (KCB_9_H_10_), assuming B_10_H_10_^2–^ and CB_9_H_10_^–^ have similar radii, the *r*_cation_/*r*_anion_ ratio, which is in the range of the 6-fold coordination packing criteria, now agrees with the observed cation coordination number. K^+^ cations in KCB_9_H_10_ are in the octahedral interstices of the *fcc*-anion sublattice with longer mean cation–anion distances than those of the tetrahedral interstitial K^+^ in K_2_B_10_H_10_. The enlarged cation coordination sphere, together with a smaller number of surrounding cations per anion required, would result in a weaker interaction between cations and anions in KCB_9_H_10_ than that in K_2_B_10_H_10_. The polarized ellipsoidal CB_9_H_10_^–^ anion with a much greater positive charge on H­(C) than its other H­(B) possesses a directional apical axis compared to the unpolarized ellipsoidal B_10_H_10_^2–^ anion. As detailed in the results section, CB_9_H_10_^–^ anions in the RT and LT1 structures can orient in certain directions so as to maximize the distances between the highly positive H­(C) atoms and the surrounding cations. In addition, the weaker cation–anion interactions are also expected between the H vertices of CB_9_H_10_^–^ anions and the surrounding cations in KCB_9_H_10_ than those in K_2_B_10_H_10_ due to the higher partial charges of H atoms in CB_9_H_10_^–^ than B_10_H_10_^2–^. Therefore, considering all of these factors combined, the fewer cations surrounding and interacting with each anion in KCB_9_H_10_ would lead to lower anion reorientational barriers, as manifested by the more rapid phase transition and more flexible anion orientation arrangement in KCB_9_H_10_ than those in K_2_B_10_H_10_ upon cooling.

Lower anion reorientational barriers and relatively more orientationally mobile anions, thus a lower phase transition temperature to the disordered HT phase, should also be expected in KCB_9_H_10_ than in K_2_B_10_H_10_ upon heating to high temperatures, which will enable more facile cation transport and higher ionic conductivity.^4,5,25^ Figure 10 also indicates reversible HT phase transitions for both K_2_B_10_H_10_ and KCB_9_H_10_, as reflected by respective enthalpic peaks centered near 692 and 548 K (during the heating segments), although the width and shape of the peaks suggest that the transitions in heating begin roughly 40 to 50 K below the peak maxima. The anion orientational flexibility observed in the ordered structures of these potassium polyborates, particularly the reduced anion reorientational barriers in KCB_9_H_10_, provides critical structural insights into the mechanisms that ultimately trigger the phase transition into highly conducting, disordered superionic states at elevated temperatures. Discussion on these HT phase transitions, which likely reflect order–disorder structural transformations, is beyond the scope of this paper. A comprehensive investigation focusing on the high-temperature disordered crystal structures, quasi-elastic neutron scattering dynamics, and electrochemical transport properties is currently underway and will be reported in a forthcoming study.

## Conclusions

Two ordered structures of K_2_B_10_H_10_ and three ordered structures of KCB_9_H_10_ were identified and determined by X-ray powder diffraction in combination with first-principles computations and corroborated by neutron vibrational spectroscopy. For K_2_B_10_H_10_, upon cooling, a monoclinic phase gradually transforms into another monoclinic phase driven by the reorientation of some of the ellipsoidal B_10_H_10_^–^ anions in the structure. With different charges and polarizabilities of the CB_9_H_10_^–^ anion and half the number of cations, KCB_9_H_10_ exhibits a more abrupt tetragonal-to-orthorhombic phase transition with the formation of two coexisting orthorhombic phases. This is also a result of the reorientation of the ellipsoidal CB_9_H_10_^–^ anions, but in a more versatile manner due to the directional apical axis of CB_9_H_10_^–^ induced by the highly positive H­(C) atoms compared to B_10_H_10_^2–^. The anion reorientational barriers in KCB_9_H_10_ are lower than those in K_2_B_10_H_10_ because of the fewer surrounding cations and weaker cation–anion interactions. Analysis on the lattice and structure changes suggests that the phase transitions in K_2_B_10_H_10_ and KCB_9_H_10_ driven by anion reorientation can lead to more efficient packing of the ellipsoidal anions and more effective bonding between cations and anions in their low-temperature structural variants. The present study provides a complete picture of the temperature-dependent ordered structural behavior for K_2_B_10_H_10_ and KCB_9_H_10_. The information on the ordered crystal structures presented here will aid in developing a better fundamental understanding of the ordered-to-disordered transformation to superionic conduction of these compounds for future studies.

## Supplementary Material



## Notes

Rietveld fit of the XRD pattern of K_2_B_10_H_10_ at 480 K, temperature-dependent synchrotron XRD patterns of K_2_B_10_H_10_, K_2_B_10_H_10_ and KCB_9_H_10_ crystal structures, and cation coordination and cation–anion distances in K_2_B_10_H_10_ and KCB_9_H_10_ (PDF)
