# Supplementary material for: Crystal Structures and Phase Evolutions of Potassium Decahydro-closo-Decaborate and Decahydro-1-Carba-closo-Decaborate: K2B10H10 and K-1-CB9H10
Source: Inorg Chem. 2026 Jul 14;65(29):16846–59. doi: 10.1021/acs.inorgchem.6c01850 (PMC13418179; doi:10.1021/acs.inorgchem.6c01850)
Supplement: Supplementary file 1 [file ic6c01850_si_001.pdf]

SUPPORTING INFORMATION

Crystal Structures and Phase Evolutions of Potassium Decahydro-*closo*-Decaborate and Decahydro-1-Carba-*closo*-Decaborate: $K_2B_{10}H_{10}$ and K-1-CB_9H_{10}

Hui Wu,[†,*] *Wei Zhou,*[†] *Benjamin A. Trump,* [†,§] *Craig M. Brown,*[†] *Terrence J. Udovic*[†,‡]

[†]NIST Center for Neutron Research, National Institute of Standards and Technology, Gaithersburg, MD 20899-6102, United States

[‡]Department of Materials Science and Engineering, University of Maryland, College Park, MD 20742, United States

[§]Intel Gordon Moore Park at Ronler Acres, Hillsboro, OR 97124, United States

*Author to whom correspondence should be addressed. E-mail: huiwu@nist.gov

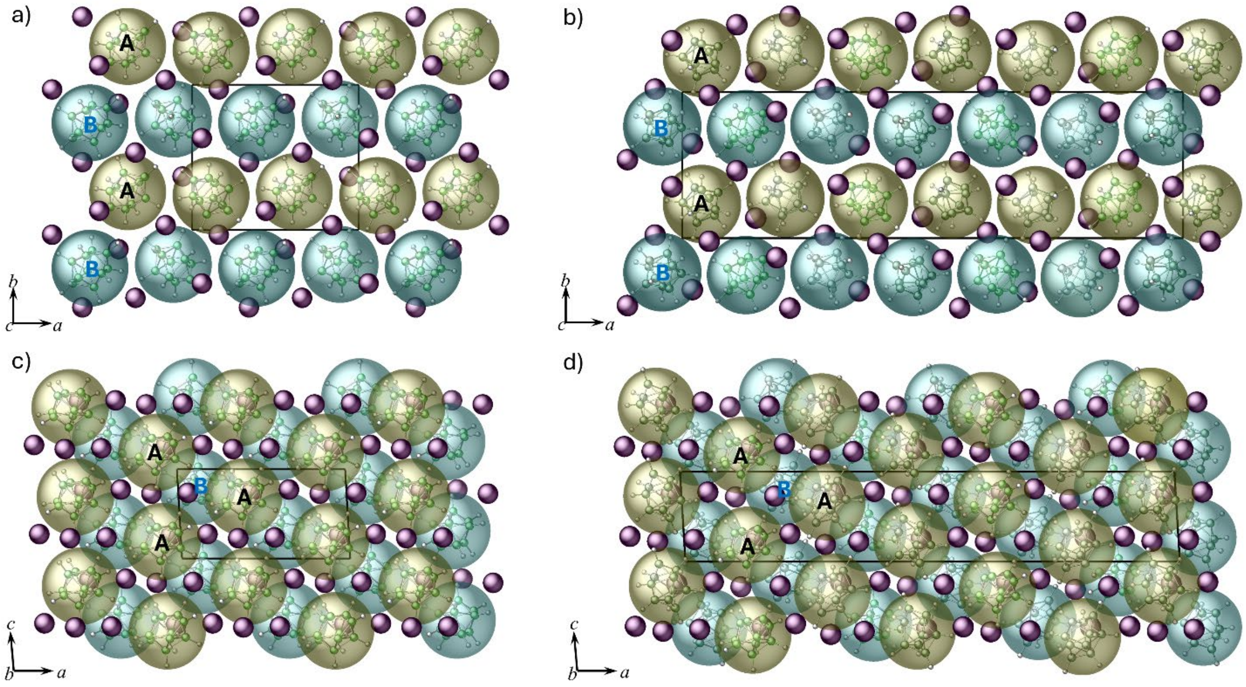

Figure S1. Comparison of the pseudo *hcp* packing arrays of $B_{10}H_{10}^{2-}$ anions and K^+ in the RT and LT structures of $K_2B_{10}H_{10}$. [001]-views of (a) RT and (b) LT structures; [010]-views of the (c) RT and (d) LT structures. $a_{LT} \approx 3a_{RT}$. Boron, hydrogen and potassium atoms are shown by bright green, white and purple solid spheres. Three crystallographically independent $B_{10}H_{10}^{2-}$ anions in the LT structure are indicated by different shades of green. The alternating positions of ABABAB… in the *hcp* arrangements of $B_{10}H_{10}^{2-}$ anion mass centers are illustrated by large transparent yellow (A) and blue (B) spheres.

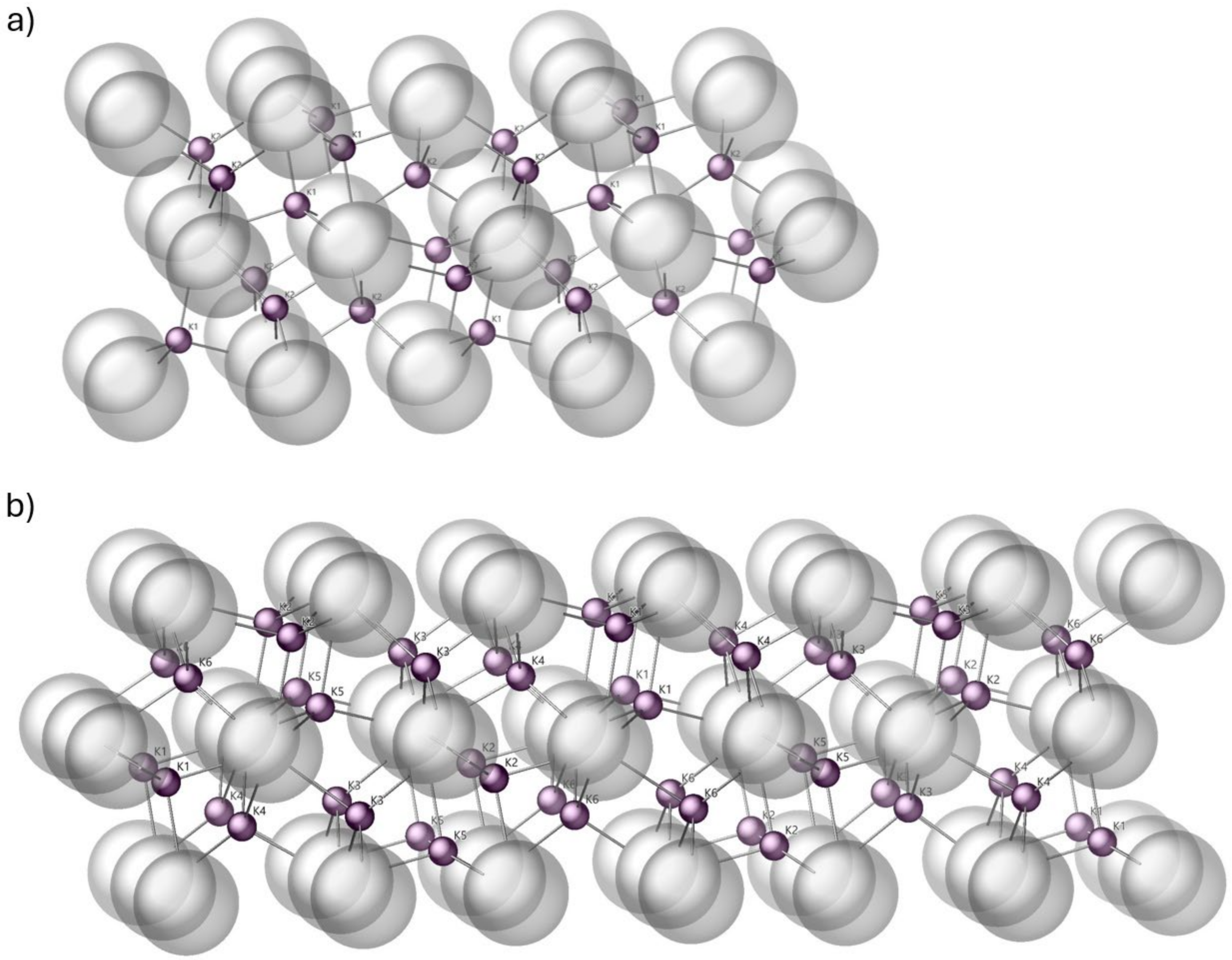

Figure S2. Anion packing scheme of $K_2B_{10}H_{10}$ and the tetrahedral interstitial positions of K^+ in the (a) RT and (b) LT structures. Potassium atoms are represented by small purple solid spheres. Mass center positions of the $B_{10}H_{10}^{2-}$ anions are illustrated by large transparent gray spheres (i.e. B and H are omitted for clarity).

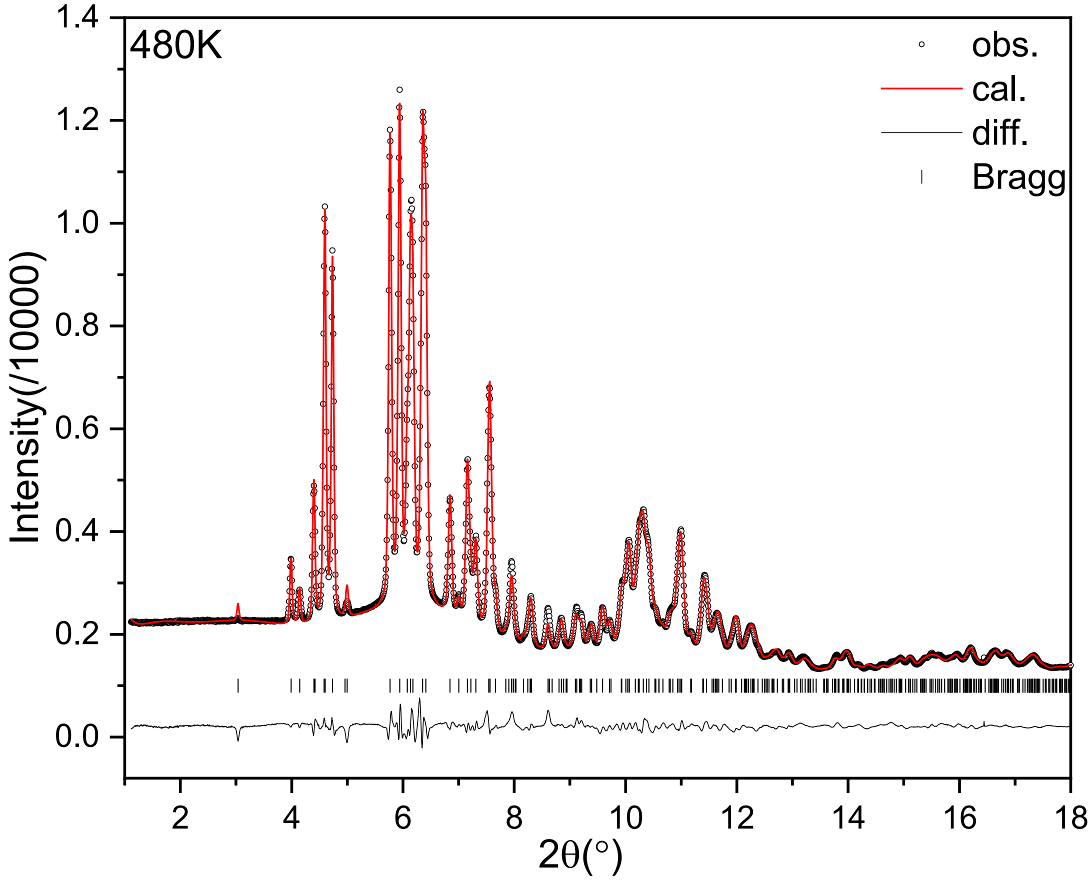

Figure S3. Experimental (circles), fitted (line), and difference (line below observed and calculated patterns) synchrotron XRD profiles for $K_2B_{10}H_{10}$ at 480 K (λ = 0.45236 Å). Vertical bars indicate the calculated positions of Bragg peaks. R_{wp}=0.0343, R_p=0.0232, χ^2=1.68. Refined lattice parameters of the monoclinic structure (S.G. *P2₁/n*): *a*=13.0271(11) Å, *b*=11.3112(10) Å, *c*=6.8764(6) Å, β=94.130(4)° and V=1010.63(23) Å^3.

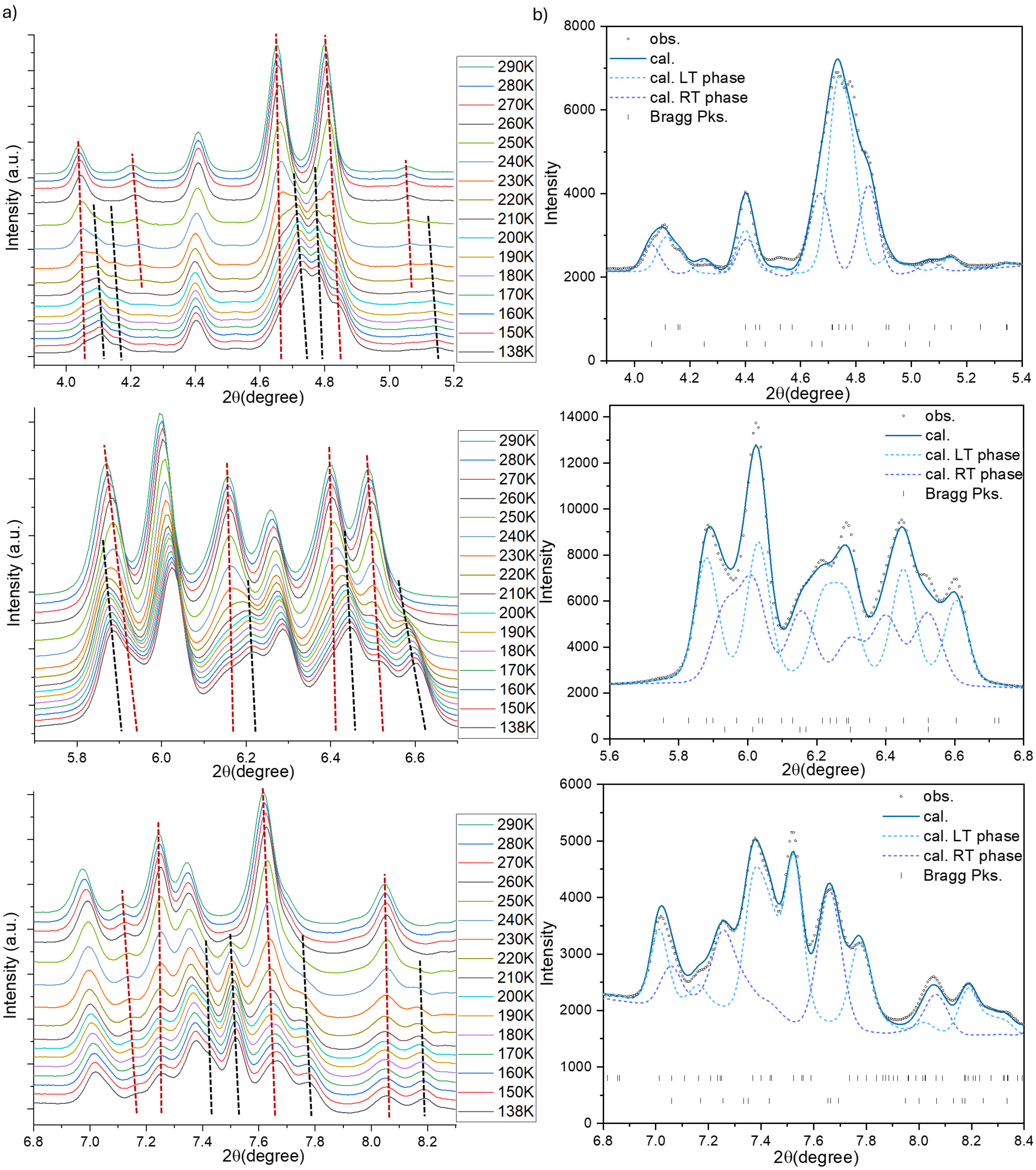

Figure S4. (a) Temperature-dependent synchrotron XRD patterns in the selected 2θ ranges of $K_2B_{10}H_{10}$. Black dashed lines indicate the emerging diffraction peaks from the LT phase, and the red dashed lines show the dwindling diffraction peaks from the RT phase with decreasing temperature; and (b) Rietveld fit of the 138 K diffraction pattern in the corresponding selected 2θ ranges shown in (a). The blue solid line, and light and dark blue dashed lines indicate the overall fit, and the contributions from the LT and RT phases, respectively. Vertical bars indicate the calculated positions of Bragg peaks of the LT phase (60.0(1) wt.%, top) and RT phase structures (40.0(1) wt.%, bottom). XRD measurement wavelength λ = 0.45236 Å.

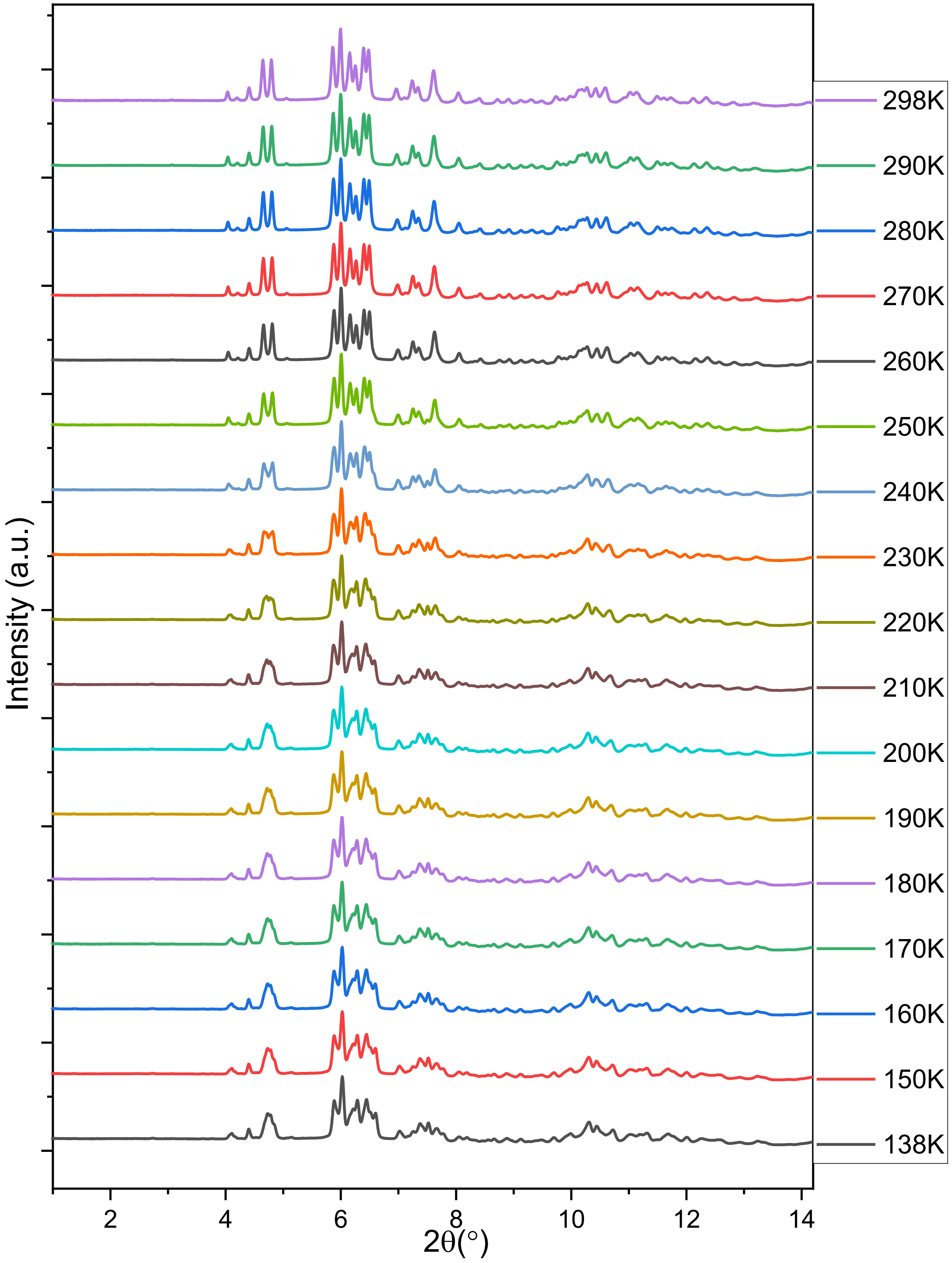

Figure S5. Temperature-dependent synchrotron XRD patterns indicating the phase evolution of $K_2B_{10}H_{10}$ from 298 K to 138 K. (XRD measurement wavelength λ = 0.45236 Å).

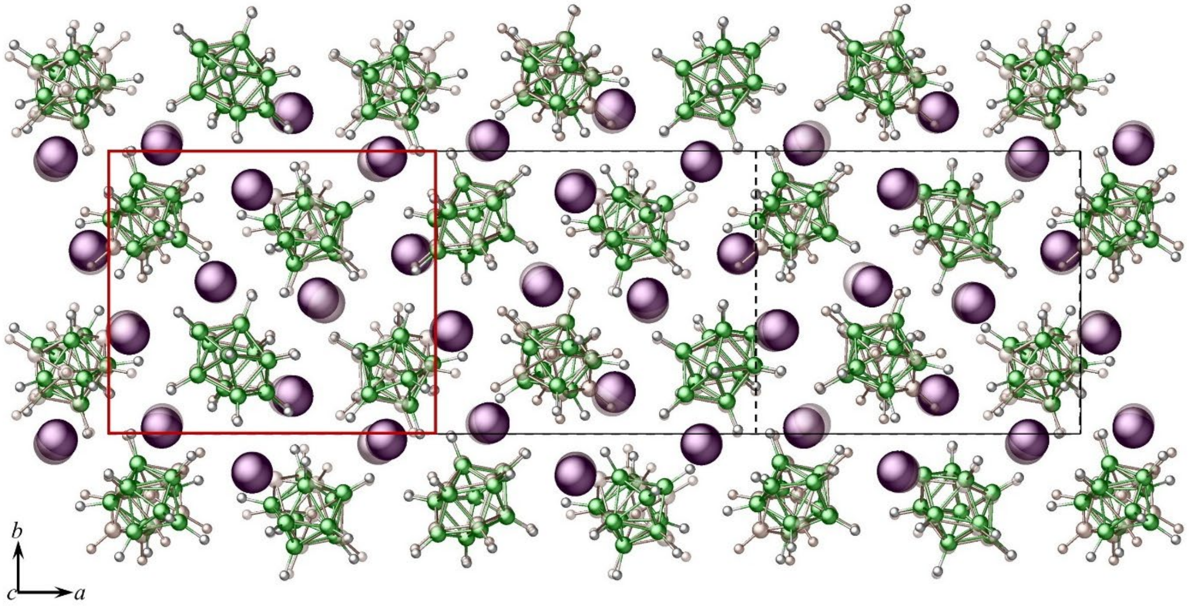

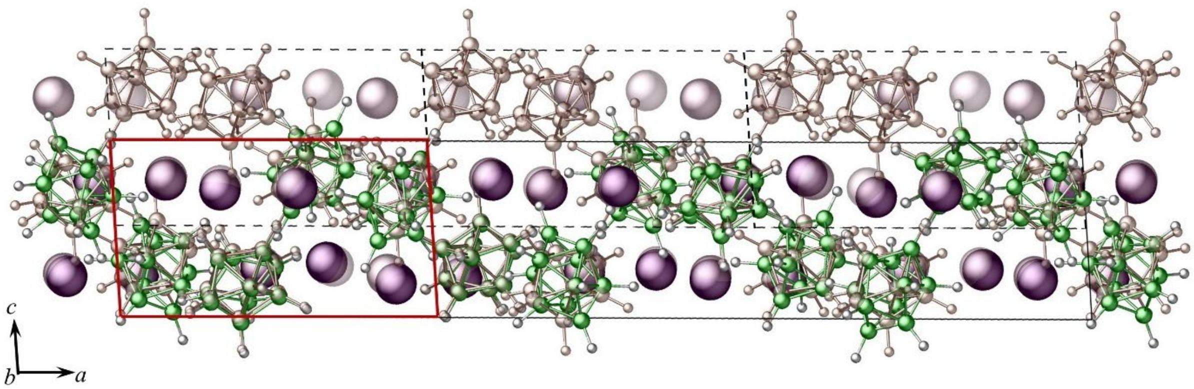

Figure S6. Overlay comparison of the arrangements of K^+ and $B_{10}H_{10}^{2-}$ in the RT and LT structures of $K_2B_{10}H_{10}$. [001] (top) and [010] (bottom) views of the LT structure overlaying the RT structure, with the RT structure *a* lattice dimension tripled (indicated in dashed lines). Red lines highlight one-third unit cell of the LT structure or one unit cell of the RT structure. Boron, hydrogen, and potassium atoms in the LT structure are represented by bright green, white and purple spheres, while these atoms are indicated in light pink colors in the RT structure.

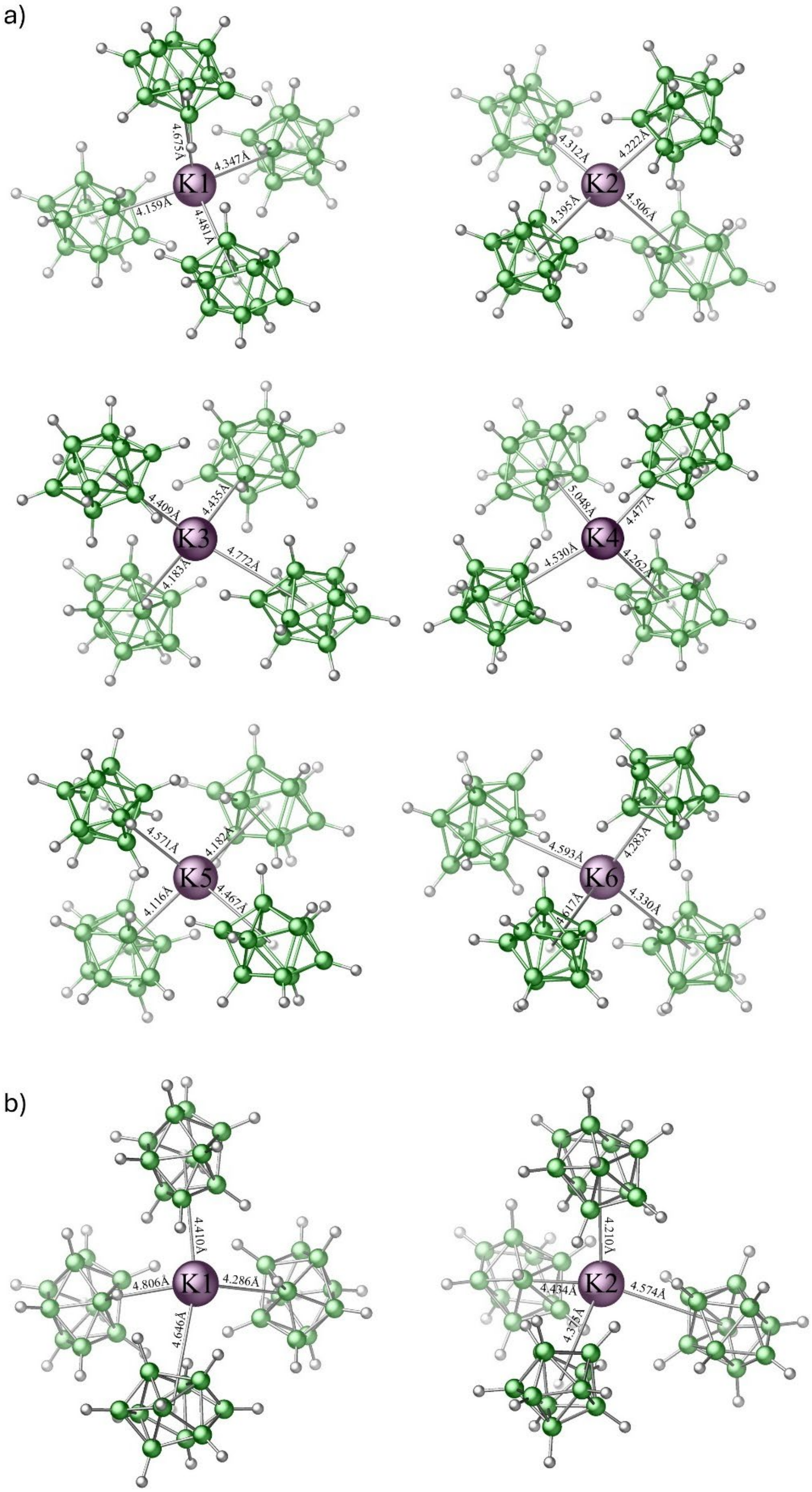

Figure S7. K coordination in (a) the LT (138 K) and (b) the RT (298 K) structures of $K_2B_{10}H_{10}$. The distances between K^+ and the mass centers of the $B_{10}H_{10}^{2-}$ anions are also shown.

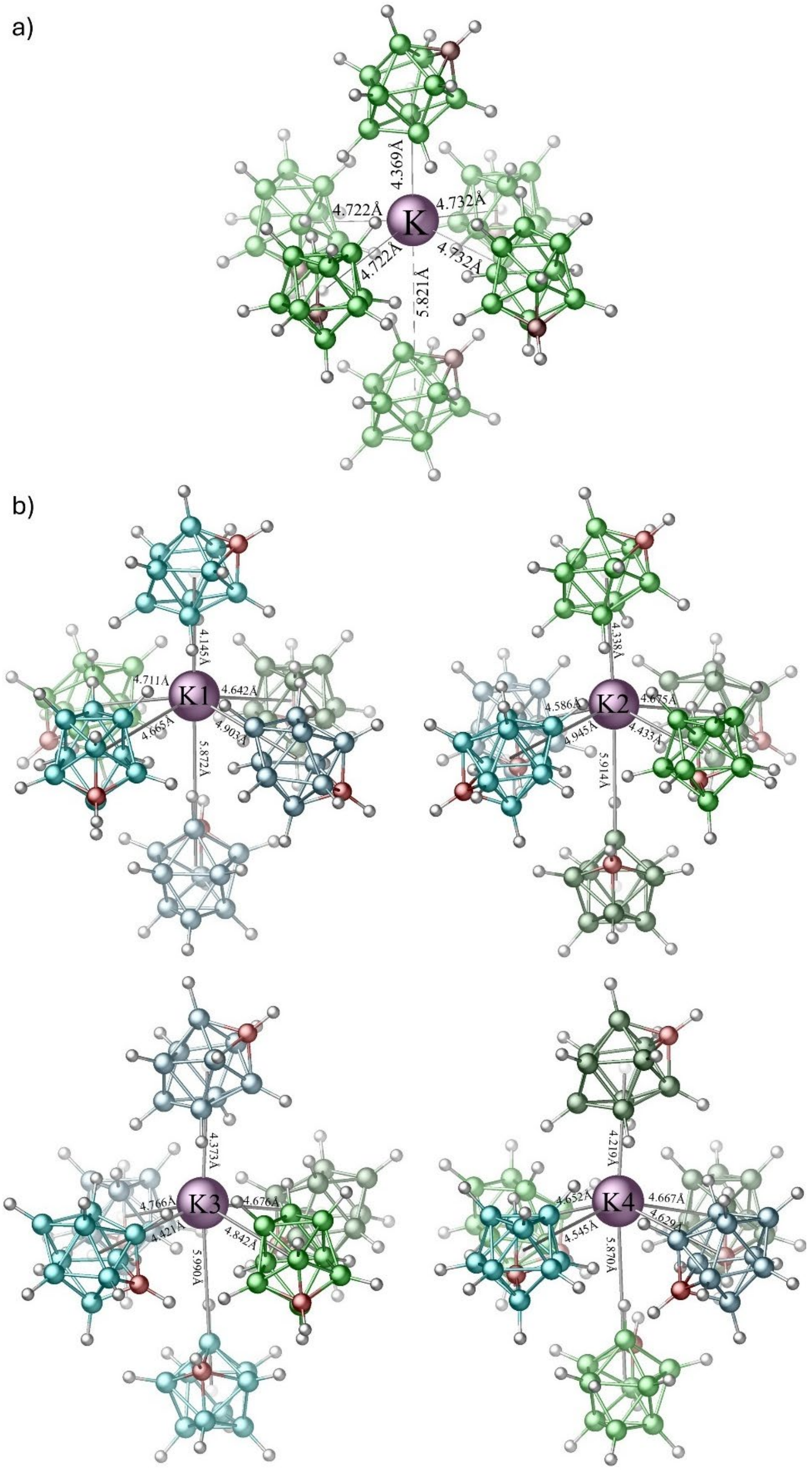

Figure S8. K coordination in (a) the RT structure (298 K) and (b) the LT1 structure (101 K) of KCB_9H_{10}. The distances between K^+ and the mass centers of the $CB_9H_{10}^-$ anions are also shown. Four crystallographically independent $CB_9H_{10}^-$ anions in the LT1 structure are indicated by bright green, cyan, dark green, light blue. C and K atoms are shown by small red and large purple solid spheres, respectively.

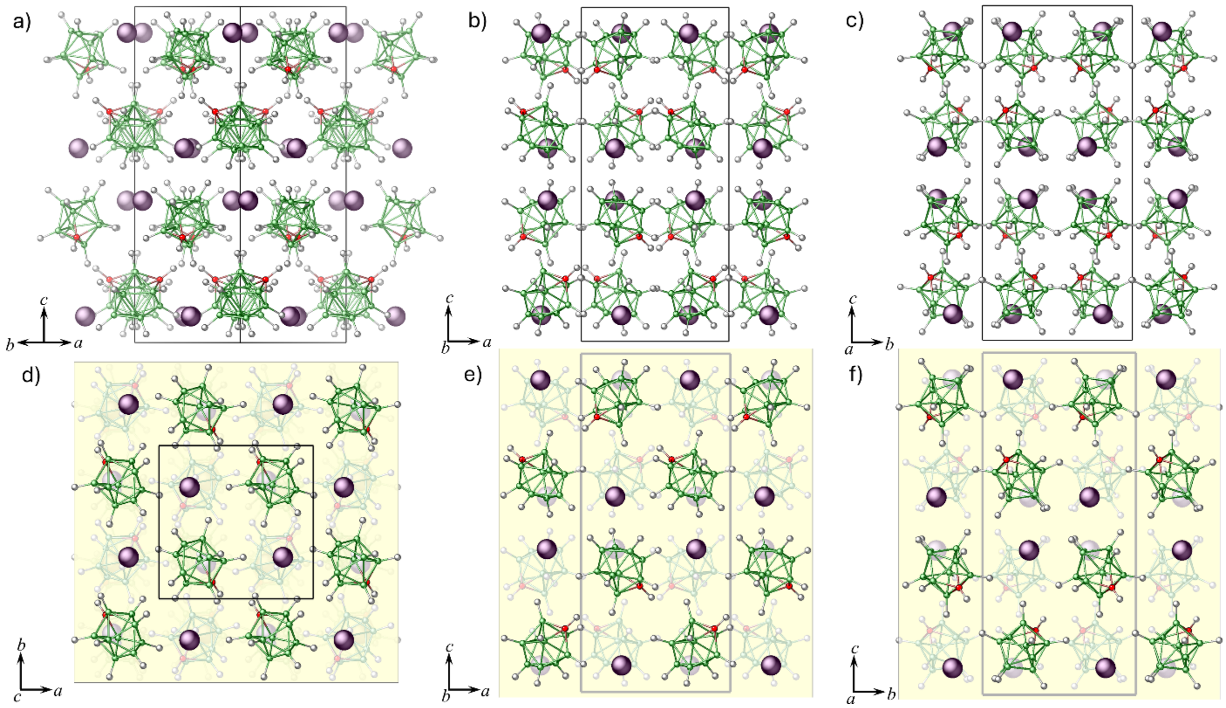

Figure S9. (a) [110]-view of the DFT-calculated orthorhombic LT2 structure model of KCB_9H_{10}. $a_{LT2}\times b_{LT2}\times c_{LT2}\approx a_{RT}\times b_{RT}\times 2c_{RT}$; (b) [010]- and (c) [100]-views of the LT2 structure with C-H apices of the $CB_9H_{10}^-$ anions in the two neighboring *ab*-planes tilting inward, forming the "H(C)-sandwiched" anion-layer pairs. K^+ cations shown by purple spheres locate between pairs of the anion layers and away from the H(C) atoms. Gray lines outline a unit cell; (d) (001)-view of the LT2 structure with one slice of $CB_9H_{10}^-$ anions shown above a (00*l*) cutting plane and an adjacent slice of anions right beneath this plane shown in faded color. The (00*l*) cutting plane is indicated by a light-yellow rectangle. Gray lines inside the light-yellow (00*l*) cutting plane outline the edges of one unit cell; (e) (010)-view of a front slice of $CB_9H_{10}^-$ anions from (b). The (0*k*0) cutting plane is indicated by a light-yellow rectangle. An adjacent slice of anions right behind this plane is also shown in faded color; and (f) (100)-view of a front slice of $CB_9H_{10}^-$ anions from (c). The (*h*00) cutting plane is indicated by a light-yellow rectangle. An adjacent slice of anions right behind this plane is also shown in faded color.

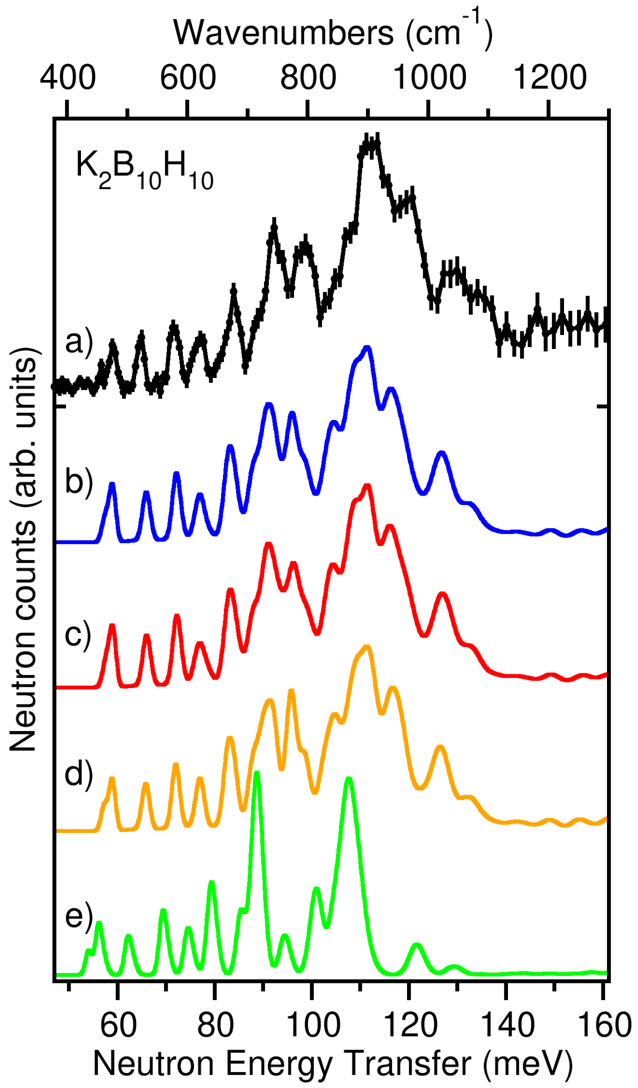

Figure S10. (a) Neutron vibrational spectrum of $K_2B_{10}H_{10}$ at 4 K compared with (b) the DFT-simulated one+two-phonon density of states representing the 60:40 weighted average of the respective monoclinic (c) LT and (d) RT structure spectra, and (e) the isolated $B_{10}H_{10}^{2-}$ anion spectrum (*N.B.*, 1 meV ≈ 8.066 cm^{-1}.)

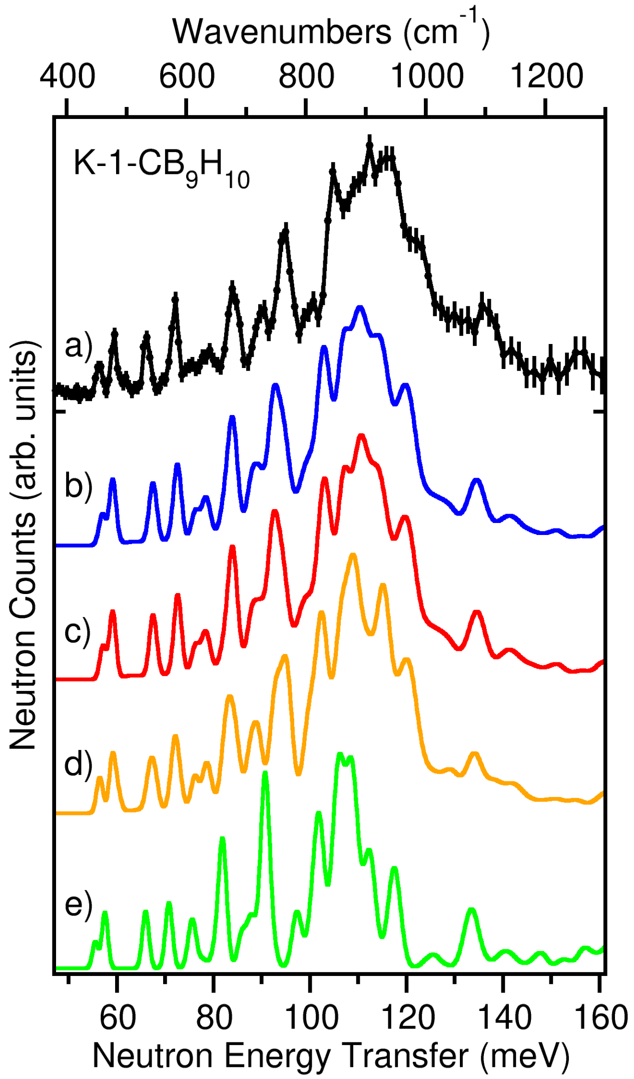

Figure S11. (a) Neutron vibrational spectrum of K-1-CB_9H_{10} at 4 K compared with (b) the DFT-simulated one+two-phonon density of states representing the 80:20 weighted average of the respective orthorhombic (c) LT1 and (d) LT2 spectra, and (e) the isolated 1-$CB_9H_{10}^-$ anion spectrum. (*N.B.*, 1 meV ≈ 8.066 cm^{-1}.)

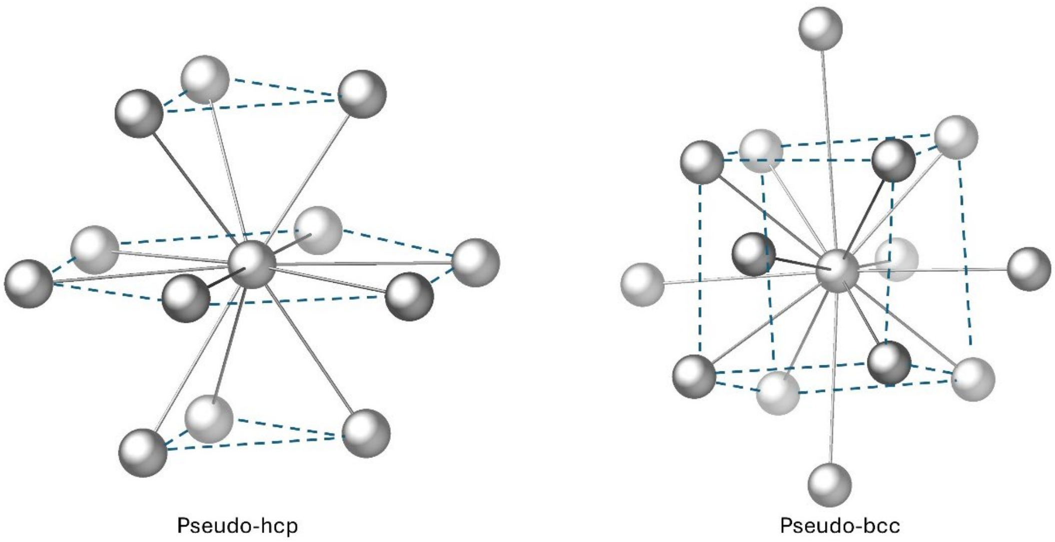

Figure S12. Packing of $B_{10}H_{10}$ anions viewed as a pseudo-*hcp* array (Left) and a distorted pseudo-*bcc* array (Right) in the RT structure of $K_2B_{10}H_{10}$. The large gray spheres represent $[B_{10}H_{10}]^{2-}$ centroids. B and H atoms are omitted for clarity. In the pseudo-*hcp* packing array (Left), the distances between the mass center of the center anion to those of its 12 neighboring anions are in the range of 6.64 Å -7.72 Å. When including two more next nearest anions with their distances to the center anion as ~8.78 Å, the anion packing can be viewed as a distorted *bcc* array with 14 surrounding anions to the center anion (Right).
